# Supplementary material for: An Assessment of the Effectiveness and Safety of Chimeric Antigen Receptor T-Cell Therapy in Multiple Myeloma Patients with Relapsed or Refractory Disease: A Systematic Review and Meta-Analysis
Source: Int J Mol Sci. 2024 May 3;25(9):4996. doi: 10.3390/ijms25094996 (PMC11084236; doi:10.3390/ijms25094996)
Supplement: Supplementary file 1 [file ijms-25-04996-s001.zip › ijms-2943606-supplementary.pdf]

Assessment of the effectiveness and safety of CAR-T cell therapy in multiple myeloma patients with relapsed or refractory disease: a systematic review and meta-analysis – Supplementary materials

Document S1: PRISMA 2020 checklist.

Section and Topic	Item #	Checklist item	Location where item is reported
**TITLE**			
Title	1	Identify the report as a systematic review.	Page 1
**ABSTRACT**			
Abstract	2	See the PRISMA 2020 for Abstracts checklist.	Page 1
**INTRODUCTION**			
Rationale	3	Describe the rationale for the review in the context of existing knowledge.	Pages 1-2
Objectives	4	Provide an explicit statement of the objective(s) or question(s) the review addresses.	Page 2-3
**METHODS**			
Eligibility criteria	5	Specify the inclusion and exclusion criteria for the review and how studies were grouped for the syntheses.	Page 3
Information sources	6	Specify all databases, registers, websites, organisations, reference lists and other sources searched or consulted to identify studies. Specify the date when each source was last searched or consulted.	Page 3
Search strategy	7	Present the full search strategies for all databases, registers and websites, including any filters and limits used.	Page 3, Table S1
Selection process	8	Specify the methods used to decide whether a study met the inclusion criteria of the review, including how many reviewers screened each record and each report retrieved, whether they worked independently, and if applicable, details of automation tools used in the process.	Page 3-4
Data collection process	9	Specify the methods used to collect data from reports, including how many reviewers collected data from each report, whether they worked independently, any processes for obtaining or confirming data from study investigators, and if	Pages 4

Section and Topic	Item #	Checklist item	Location where item is reported
		applicable, details of automation tools used in the process.	
Data items	10a	List and define all outcomes for which data were sought. Specify whether all results that were compatible with each outcome domain in each study were sought (e.g. for all measures, time points, analyses), and if not, the methods used to decide which results to collect.	Page 4
	10b	List and define all other variables for which data were sought (e.g. participant and intervention characteristics, funding sources). Describe any assumptions made about any missing or unclear information.	Page 4
Study risk of bias assessment	11	Specify the methods used to assess risk of bias in the included studies, including details of the tool(s) used, how many reviewers assessed each study and whether they worked independently, and if applicable, details of automation tools used in the process.	Page 4
Effect measures	12	Specify for each outcome the effect measure(s) (e.g. risk ratio, mean difference) used in the synthesis or presentation of results.	Page 4
Synthesis methods	13a	Describe the processes used to decide which studies were eligible for each synthesis (e.g. tabulating the study intervention characteristics and comparing against the planned groups for each synthesis (item #5)).	Pages 4
	13b	Describe any methods required to prepare the data for presentation or synthesis, such as handling of missing summary statistics, or data conversions.	Page 4
	13c	Describe any methods used to tabulate or visually display results of individual studies and syntheses.	Page 4
	13d	Describe any methods used to synthesize results and provide a rationale for the choice(s). If meta-analysis was performed, describe the model(s), method(s) to identify the presence and extent of statistical heterogeneity, and software package(s) used.	Page 4
	13e	Describe any methods used to explore possible causes of heterogeneity among study results (e.g. subgroup analysis, meta-regression).	Page 4
	13f	Describe any sensitivity analyses conducted to assess robustness of the synthesized results.	Not applicable
Reporting bias assessment	14	Describe any methods used to assess risk of bias due to missing results in a synthesis (arising from reporting biases).	Page 4
Certainty	15	Describe any methods used to assess certainty (or confidence) in the body of evidence for an outcome.	Page 4

Section and Topic	Item #	Checklist item	Location where item is reported
assessment			
**RESULTS**			
Study selection	16a	Describe the results of the search and selection process, from the number of records identified in the search to the number of studies included in the review, ideally using a flow diagram.	Page 5, Figure 1
	16b	Cite studies that might appear to meet the inclusion criteria, but which were excluded, and explain why they were excluded.	Figure 1
Study characteristics	17	Cite each included study and present its characteristics.	Pages 5-6, Tables S2-4 and 1-2
Risk of bias in studies	18	Present assessments of risk of bias for each included study.	Page 20-21, Table S5
Results of individual studies	19	For all outcomes, present, for each study: (a) summary statistics for each group (where appropriate) and (b) an effect estimate and its precision (e.g. confidence/credible interval), ideally using structured tables or plots.	Pages 14-17, Figures S1-2 and 2-8
Results of syntheses	20a	For each synthesis, briefly summarise the characteristics and risk of bias among contributing studies.	Page 20-21, Table S5
	20b	Present results of all statistical syntheses conducted. If meta-analysis was done, present for each the summary estimate and its precision (e.g. confidence/credible interval) and measures of statistical heterogeneity. If comparing groups, describe the direction of the effect.	Pages 14-17, Figures S1-2, and 2-8
	20c	Present results of all investigations of possible causes of heterogeneity among study results.	Page 17, Table 3, Figures S3 and 9-12
	20d	Present results of all sensitivity analyses conducted to assess the robustness of the synthesized results.	Not applicable
Reporting biases	21	Present assessments of risk of bias due to missing results (arising from reporting biases) for each synthesis assessed.	Page 20-21, Figure 13
Certainty of	22	Present assessments of certainty (or confidence) in the body of evidence for each outcome assessed.	Figures S1-3

Section and Topic	Item #	Checklist item	Location where item is reported
evidence			and 2-8
**DISCUSSION**			
Discussion	23a	Provide a general interpretation of the results in the context of other evidence.	Pages 21-24
	23b	Discuss any limitations of the evidence included in the review.	Pages 21-24
	23c	Discuss any limitations of the review processes used.	Pages 21-24
	23d	Discuss implications of the results for practice, policy, and future research.	Pages 21-24
**OTHER INFORMATION**			
Registration and protocol	24a	Provide registration information for the review, including register name and registration number, or state that the review was not registered.	Not applicable
	24b	Indicate where the review protocol can be accessed, or state that a protocol was not prepared.	Not applicable
	24c	Describe and explain any amendments to information provided at registration or in the protocol.	Not applicable
Support	25	Describe sources of financial or non-financial support for the review, and the role of the funders or sponsors in the review.	Page 25
Competing interests	26	Declare any competing interests of review authors.	Page 25
Availability of data, code and other materials	27	Report which of the following are publicly available and where they can be found: template data collection forms; data extracted from included studies; data used for all analyses; analytic code; any other materials used in the review.	Page 25

Table S1. Search strategy.

Database	Search query	Total results
**PubMed**	(“multiple myeloma” [MeSH Terms] OR "multiple myeloma"[Title/Abstract] OR "relapsed multiple myeloma"[Title/Abstract] OR "refractory multiple myeloma"[Title/Abstract]) AND ("immunotherapy, adoptive"[MeSH Terms] OR "car t cell"[Title/Abstract] OR "chimeric antigen receptor T-cell therapy"[Title/Abstract] OR "car t immunotherapy"[Title/Abstract])	705
**Scopus**	TITLE-ABS-KEY (("multiple myeloma" OR "relapsed multiple myeloma" OR "refractory multiple myeloma") AND ("immunotherapy, adoptive" OR "car t cell" OR "chimeric antigen receptor T-cell therapy" OR "car t immunotherapy"))	974
**Web of Science**	(TI=("multiple myeloma" OR "relapsed multiple myeloma" OR "refractory multiple myeloma") OR AB=(“multiple myeloma" OR "relapsed multiple myeloma" OR "refractory multiple myeloma") OR KP=("multiple myeloma" OR "relapsed multiple myeloma" OR "refractory multiple myeloma")) AND (TI= ("immunotherapy, adoptive" OR "car t cell" OR "chimeric antigen receptor T-cell therapy" OR "car t immunotherapy") OR AB= ("immunotherapy, adoptive" OR "car t cell" OR "chimeric antigen receptor T-cell therapy" OR "car t immunotherapy") OR KP= ("immunotherapy, adoptive" OR "car t cell" OR "chimeric antigen receptor T-cell therapy" OR "car t immunotherapy"))	594

Table S2. Characteristics of the included studies.

First author	Publication year	Study design	Production name	Registration number	Study phase	Country	Sample size
**Raje**	2019	Prospective study (clinical trial)	Idecabtagene vicleucel/ide-cel/bb2121, (CRB-401)	NCT02658929	Phase 1	USA	33
**Xu**	2019	Prospective study (clinical trial)	LCAR-B38M	NCT03090659 ChiCTR-ONH-17012285	Phase 1	China	17
**Cohen**	2019	Prospective study (clinical trial)	CART-BCMA	NCT02546167	Phase 1	USA	25
**Yan**	2020	Prospective study (clinical trial)	CD19 & BCMA CAR-T	NCT03196414	Phase 1	China	10
**Chen**	2020	Prospective study (clinical trial)	CD19 & BCMA CAR-T cells	ChiCTROIC-17011272	Phase 2	China	7
**Wang**	2021	Prospective study (clinical trial)	CT103A	ChiCTR1800018137	Phase 1	China	18
**Cornell**	2021	Prospective study (clinical trial)	KITE-585 CAR	NCT03318861	Phase 1	USA	14
**Mei**	2021	Prospective study (clinical trial)	BM38 CAR	ChiCTR1800018143	Phase 1	China	23
**Munshi**	2021	Prospective study (clinical trial)	idecabtagene vicleucel/ ide-cel/ bb2121 (KarMMa)	NCT03361748	Phase 2	USA, Canada, Belgium, France, Germany, Italy, Japan, Spain	128
**Zhang**	2022	Prospective study (clinical trial)	BCMA & CD38 CAR-T cells	ChiCTR1800017051	Phase 2	China	22
**Du**	2022	Prospective study (clinical trial)	BCMA CAR/ HDS269B	NCT03093168	Phase 1/2	China	49

**Wang**	2022	Prospective study (clinical trial)	CD19 & BCMA CAR-T cells	ChiCTROIC-17011272	Phase 2	China	62
**Zhao**	2022	Prospective study (clinical trial)	LCAR-B38M (LEGEND-2)	NCT03090659 ChiCTR-ONH-17012285	Phase 1	China	74
**Mailankody**	2022	Prospective study (clinical trial)	GPRC5D CAR-T cells/ MCARH109	NCT04555551	Phase 1	USA	17
**Qu**	2022	Prospective study (clinical trial)	C-CAR088	NCT03815383 NCT03751293 NCT04295018 NCT04322292	Phase 1	China	31
**Tang**	2022	Prospective study (clinical trial)	BCMA & CD38 CAR-T cells	ChiCTR1900026286	Phase 1	China	16
**Ri**	2022	Prospective study (clinical trial)	Ciltacabtagene autoleucel/cilta-cel/ JNJ-68284528 (CARTITUDE-1)	NCT03548207	Phase 2	Japan	9
**Martin**	2022	Prospective study (clinical trial)	Ciltacabtagene autoleucel/cilta-cel/ JNJ-68284528 (CARTITUDE-1)	NCT03548207	Phase 1b/2	USA	97
**Mi**	2022	Prospective study (clinical trial)	Ciltacabtagene autoleucel/cilta-cel/ JNJ-68284528 (CARTIFAN-1)	NCTO3758417	Phase 2	China	48
**Asherie**	2022	Prospective study (clinical trial)	HBI0101	NCT04720313	Phase 1	Israel	20
**Cohen**	2023	Prospective study (clinical trial)	Ciltacabtagene autoleucel/cilta-cel/ JNJ-68284528 (CARTITUDE-2, cohort C)	NCT04133636	Phase 2	USA, Belgium, France, Germany, Israel, Netherlands, Saudi Arabia, Spain	20
**Xia**	2023	Prospective study (clinical trial)	GPRC5D CAR-T cells	ChiCTR2100048888	Phase 2	China	33
**Mailankody**	2023	Prospective study (clinical trial)	ALLO-715 (UNIVERSAL, cohort A)	NCT04093596	Phase 1	USA	43

**Lee**	2023	Prospective study (clinical trial)	APRIL CAR-T cells (AUTO2)	NCT03287804	Phase 1	UK, Netherlands	11
**Oliver-Caldés**	2023	Prospective study (clinical trial)	ARI0002h (CARTBCMA-HBC-01)	NCT04309981	Phase 1/2	Spain	30
**Zhang**	2023	Prospective study (clinical trial)	GPRC5D CAR-T cells/ OriCAR-017 (POLARIS)	NCT05016778	Phase 1	China	10
**Minakata**	2023	Prospective study (clinical trial)	idecabtagene vicleucel/ ide-cel/ bb2121, (KarMMa)	NCT03361748	Phase 2	Japan	9
**Sanoyan**	2023	Retrospective study (real-world data)	idecabtagene/ide-cel/bb2121	NA	NA	Switzerland	16
**Hansen**	2023	Retrospective study (real-world data)	idecabtagene/ide-cel/ bb2121	NA	NA	USA	159

NA: Not Applicable; NR: Not Reported.

Table S3. Continued: Characteristics of the included studies.

First author, year	Target antigen	T cell source	Gene transfer method	Costimulatory domain	scFv species	CAR-T cell generation
**Raje 2019**	BCMA	Autologous	Lentivirus	4-1BB	Murine	Second-generation
**Xu 2019**	BCMA	Autologous	Lentivirus	4-1BB	Llama	Second-generation
**Cohen 2019**	BCMA	Autologous	Lentivirus	4-1BB	Human	Second-generation
**Yan 2020**	BCMA, CD19	Autologous Allogeneic	Lentivirus	4-1BB/OX40	Humanized	Third-generation
**Chen 2020**	BCMA, CD19	Autologous	Lentivirus	4-1BB	Anti-CD19 - Humanized Anti-BCMA - Murine	Third-generation
**Wang 2021**	BCMA	Autologous	Lentivirus	4-1BB	Human	Second-generation
**Cornell 2021**	BCMA	Autologous	Lentivirus	CD28	Human	Second-generation
**Mei 2021**	BCMA, CD38	Autologous	Lentivirus	4-1BB	Anti-BCMA - Human Anti-CD38 - Humanized	Second-generation
**Munshi 2021**	BCMA	Autologous	Lentivirus	4-1BB	Murine	Second-generation
**Zhang 2022**	BCMA, CD38	Autologous	Lentivirus	4-1BB	Anti-BCMA - Humanized Anti-CD38 - Murine	Second-generation
**Du 2022**	BCMA	Autologous	Retrovirus	4-1BB	Murine	Second-generation
**Wang 2022**	BCMA, CD19	Autologous	Lentivirus	4-1BB	Anti-CD19 – Humanized Anti-BCMA – Murine	Third-generation

**Zhao 2022**	BCMA	Autologous	Lentivirus	4-1BB	Llama	Second-generation
**Mailankody 2022**	GPRC5D	Autologous	Lentivirus	4-1BB	Human	Second-generation
**Qu 2022**	BCMA	Autologous	Lentivirus	4-1BB	Human	Second-generation
**Tang 2022**	BCMA, CD38	Autologous	Lentivirus	4-1BB	NR	Second-generation
**Ri 2022**	BCMA	Autologous	Lentivirus	4-1BB	Llama	Second-generation
**Martin 2022**	BCMA	Autologous	Lentivirus	4-1BB	Llama	Second-generation
**Mi 2022**	BCMA	Autologous	Lentivirus	4-1BB	Llama	Second-generation
**Asherie 2022**	BCMA	Autologous	Retrovirus	4-1BB	Murine	Second-generation
**Cohen 2023**	BCMA	Autologous	Lentivirus	4-1BB	Llama	Second-generation
**Xia 2023**	GPRC5D	Autologous	Lentivirus	4-1BB	Human	Second-generation
**Mailankody 2023**	BCMA	Allogeneic	Lentivirus	4-1BB	Human	Second-generation
**Lee 2023**	BCMA, TACI	Autologous	Retrovirus	CD28, OX40	NR	Third-generation
**Oliver-Caldés 2023**	BCMA	Autologous	Lentivirus	4-1BB	Humanized	Second-generation
**Zhang 2023**	GPRC5D	Autologous	Lentivirus	4-1BB	Llama	Second-generation

**Minakata 2023**	BCMA	Autologous	Lentivirus	4-1BB	Murine	Second-generation
**Sanoyan 2023**	BCMA	Autologous	Lentivirus	4-1BB	Murine	Second-generation
**Hansen 2023**	BCMA	Autologous	Lentivirus	4-1BB	Murine	Second-generation

NR: Not Reported.

Table S4. Initial qualities of the enrolled participants.

First author, year	Age, median (range), y	Male/female	Race	Median time since diagnosis (range)	ECOG PS score, number (%)	ISS staging, number (%)
**Raje 2019**	60 (37-75)	21/12	NR	5y (1-36)	0 – 10 (30)<br>1 – 21 (64)<br>2 – 2 (6)	I – 7 (21)<br>II – 14 (42)<br>III – 8 (24)<br>Unknown – 4 (12)
**Xu 2019**	55 (35-73)	11/6	NR	NR	NR	NR
**Cohen 2019**	58 (44-75)	17/8	NR	4.6y (1.8-14.5)	NR	NR
**Yan 2020**	56 (43-69)	7/3	NR	NR	NR	NR
**Chen 2020**	49 (41-55)	7/0	NR	23.5m (7.9-34.2)	NR	I – 2 (29)<br>II – 2 (29)<br>III – 3 (43)
**Wang 2021**	53.5 (38-66)	10/8	NR	31.9m (8.80-94.3)	0 – 3 (17)<br>1 – 15 (83)	I – 10 (56)<br>II – 8 (44)<br>III – 0
**Cornell 2021**	56 (47-71)	10/7	NR	61m (27-134)	1 – 7 (41)	I/II – 7 (41)<br>III – 4 (24)<br>Unknown – 6 (35)
**Mei 2021**	59 (49-72)	11/12	NR	2.9y (0.4-13.4)	0-2 – 19 (83)<br>3-4 – 4 (17)	I – 6 (26)<br>II – 6 (26)<br>III – 11 (48)
**Munshi 2021**	61 (33-78)	76/52	NR	6y (1-18)	0 – 57 (45)<br>1 – 68 (53)<br>2 – 3 (2)	I – 14 (11)<br>II – 90 (70)<br>III – 21 (16)<br>Unknown – 3 (2)

**Zhang 2022**	56 (47-68)	9/13	NR	NR	NR	I – 0 (0)<br>II – 17 (77.3)<br>III – 5 (22.7)
**Du 2022**	57 (37-75)	26/23	NR	2.7y (0.3-12.6)	0/1 – 17 (34.69)<br>2 – 12 (22.49)<br>3 – 14 (28.57)<br>4 – 6 (12.25)	I – 6 (12.24)<br>II – 20 (40.82)<br>III – 13 (26.53)<br>Unknown – 10 (20.41)
**Wang 2022**	58 (30-69)	34/28	NR	30m (8-167)	NR	I – 8 (13)<br>II – 24 (39)<br>III – 30 (48)
**Zhao 2022**	54.5 (27-74)	45/29	NR	4.0y (1-9)	0 – 30 (40.5)<br>1 – 32 (43.2)<br>2 – 12 (16.2)	I – 33 (44.6)<br>II – 14 (18.9)<br>III – 21 (28.4)<br>Unknown – 6 (8.1)
**Mailankody 2022**	60 (38-76)	13/4	NR	NR	NR	NR
**Qu 2022**	61 (45-74)	17/14	NR	NR	0 – 19 (61.3)<br>1 – 12 (38.7)	I – 4 (12.9)<br>II – 21 (67.7)<br>III – 5 (16.2)<br>Unknown – 1 (3.2)
**Tang 2022**	58.5 (48-78)	NR	NR	28.5m (4-72)	NR	I – 2 (12.5)<br>II – 1 (6.25)<br>III – 13 (81.3)
**Ri 2022**	57 (45-71)	5/4	Asian, 9 (100)	5.41y (3.8-11.3)	0 – 7 (77.8)<br>1 – 1 (11.1)<br>2 – 1 (11.1)	I – 5 (55.6)<br>II – 3 (33.3)<br>III – 1 (11.1)
**Martin 2022**	61 (56-68)	57/40	White, 69 (71); Black or African American, 17 (18); Asian, 1 (1); American Indian or Alaska native, 1(1); Native	5.9y (4.4-8.4)	0 – 39 (40)<br>1 – 54 (56)<br>2 – 4 (4)	I – 61 (63)<br>II – 22 (23)<br>III – 14 (14)

			Hawaiian or other Pacific Islander, 1(1); Not reported, 8 (8)			
**Mi 2022**	61 (30-72)	32/16	NR	3.7y (1.4-10.2)	0 – 22 (45.8)<br>1 – 26 (54.2)	I – 21 (43.8)<br>II – 18 (37.5)<br>III – 9 (18.8)
**Asherie 2022**	62 (44-75)	8/12	NR	55m (8-241)	0 – 7 (35)<br>1 – 4 (20)<br>2 – 9 (45)	I – 1 (5)<br>II – 11 (55)<br>III – 2 (10)
**Cohen 2023**	62.5 (44-81)	12/8	White, 19 (95); Black, 1 (5)	6.3y (2.5-16.3)	0 – 8 (40)<br>1 – 12 (60)	I– 8 (40)<br>II – 4 (20)<br>III – 8 (40)
**Xia 2023**	58 (39-70)	18/15	NR	31.5m (4.8-96.0)	NR	I – 6 (18)<br>II – 15 (45)<br>III – 12 (36)
**Mailankody 2023**	64 (46-77)	27/16	White, 35 (81.4); Black or African American, 5 (11.6); Asian, 2 (4.7); Native Hawaiian or Pacific Islander, 1(2.3)	4.9y (0.9-26.4)	0 – 21 (48.8)<br>1 – 22 (51.2)	I – 12 (27.9)<br>II – 22 (51.2)<br>III – 8 (18.6)<br>Unknown – 1 (2.3)
**Lee 2023**	61 (45-69)	8/3	NR	6y (1-11)	NR	I – 6 (54.5)<br>II – 1 (9.1)<br>III – 3 (27.3)<br>Unknown – 1 (9.1)
**Oliver-Caldés 2023**	61 (53-65)	18/12	NR	4.7y (3.7-9.1)	0 – 18/29 (62)<br>1 – 9/29 (31)<br>2 – 2/29 (7)	I – 5/25 (20)<br>II – 8/25 (32)<br>III – 12/25 (48)

**Zhang 2023**	64 (58-68)	5/5	Chinese, 10 (100)	39m (25-78)	0 – 1 (10)<br>1 – 3 (30)<br>2 – 6 (60)	I – 2 (20)<br>II – 5 (50)<br>III – 3 (30)
**Minakata 2023**	54 (38-73)	7/2	Asian, 9 (100)	3.6y (1.0-7.9)	0 – 6 (67)<br>1 – 3 (33)<br>2 – 0	I – 2 (22)<br>II – 4 (44)<br>III – 2 (22)<br>Unknown – 1 (11)
**Sanoyan 2023**	69 (57-83)	11/5	NR	7.7y (2.1-16.7)	0 – 11 (69)<br>1 – 4 (25)<br>2 – 1 (6)	I – 6 (38)<br>II – 7 (44)<br>III – 3 (19)
**Hansen 2023**	64 (36-83)	91/68	NR	NR	0-1 – 127 (81)<br>2-4 – 29 (19)<br>Unknown – 31	I – 22 (17)<br>II – 71 (55)<br>III – 35 (27)<br>Unknown – 31

NR: Not Reported.

Table S5. MINORS – Quality Assessment.

Study	MINORS EVALUATION								Total
	I	II	III	IV	V	VI	VII	VII	
**Raje 2019**	2	2	2	2	2	2	2	0	14
**Xu 2019**	2	2	2	1	1	2	2	0	12
**Cohen 2019**	2	2	2	2	2	0	2	0	12
**Yan 2020**	2	2	2	2	1	2	2	0	13
**Chen 2020**	2	2	2	1	1	2	2	0	12
**Wang 2021**	2	2	2	2	1	1	2	0	12
**Cornell 2021**	2	2	2	2	1	2	2	0	13
**Mei 2021**	2	2	2	2	2	2	2	0	14
**Munshi 2021**	2	2	2	2	2	2	2	2	16
**Zhang 2022**	2	2	2	2	2	2	2	0	14
**Du 2022**	2	2	2	2	2	2	2	0	14
**Wang 2022**	2	2	2	2	2	2	2	0	14
**Zhao 2022**	2	2	2	2	2	2	2	0	14
**Mailankody 2022**	2	2	2	2	1	2	2	0	13
**Qu 2022**	2	2	2	2	2	2	2	0	14
**Tang 2022**	2	2	2	2	1	2	2	0	13
**Ri 2022**	2	2	2	2	1	1	2	2	14
**Martin 2022**	2	2	2	2	2	2	2	2	16
**Mi 2022**	2	2	2	2	2	2	2	2	16
**Asherie 2022**	2	2	2	2	1	1	2	2	14
**Cohen 2023**	2	2	2	2	1	2	2	0	13
**Xia 2023**	2	2	2	2	2	1	2	0	13
**Mailankody 2023**	2	2	2	1	2	2	2	0	13
**Lee 2023**	2	2	2	2	1	0	2	2	13
**Oliver-Caldés 2023**	2	2	2	2	2	2	2	0	14
**Zhang 2023**	2	2	2	2	1	1	2	0	12
**Minakata 2023**	2	2	2	2	1	2	2	2	15
**Sanoyan 2023**	2	2	1	2	1	1	2	0	11
**Hansen 2023**	2	2	1	2	1	1	2	0	11

I, A clearly stated aim; II, Inclusion of consecutive patients; III, Prospective collection of data; IV, Endpoints appropriate to the aim of the study; V, Unbiased assessment of the study endpoint; VI, Follow-up period appropriate to the aim of the study; VII, Loss to follow up less than 5%; VIII, Prospective calculation of the study size.

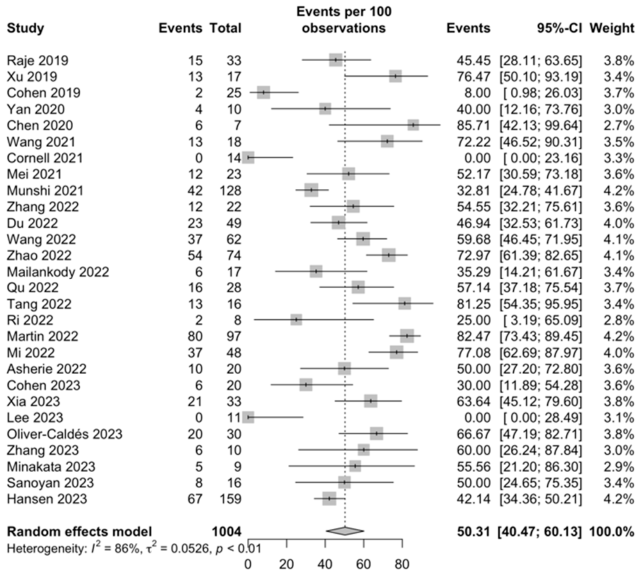

(A)

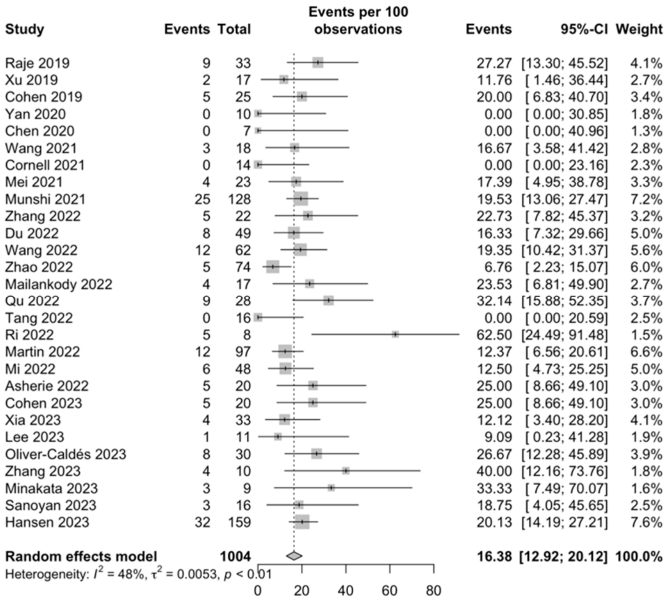

(B)

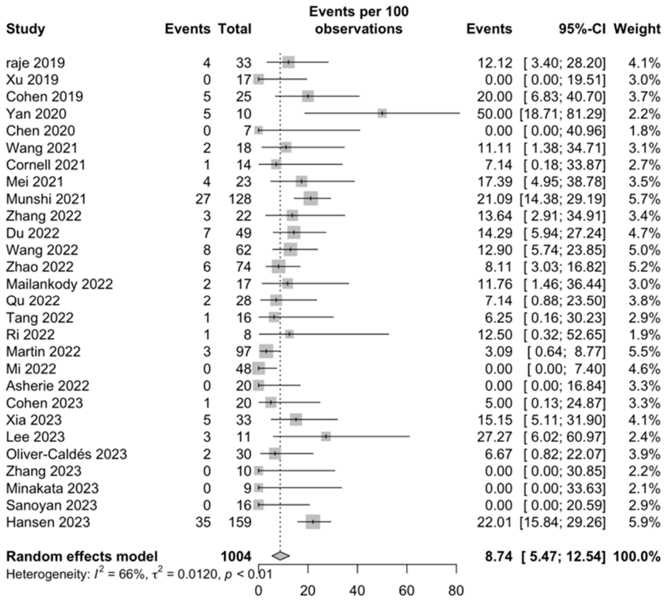

(C)

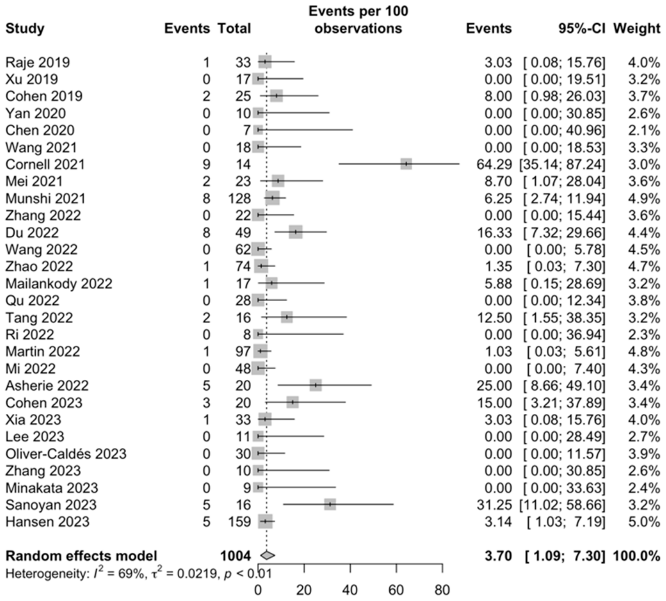

(D)

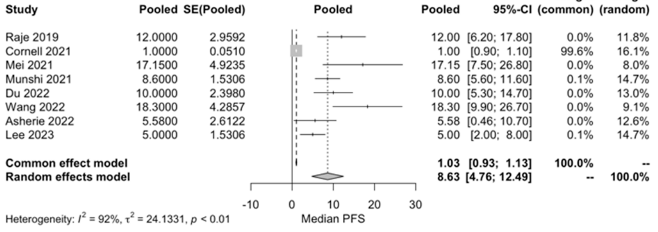

(E)

Figure S1. Efficacy outcomes: (**A**) CRR; **(B)** vgPR; **(C)** PR; **(D)** PD; **(E)** mPFS.

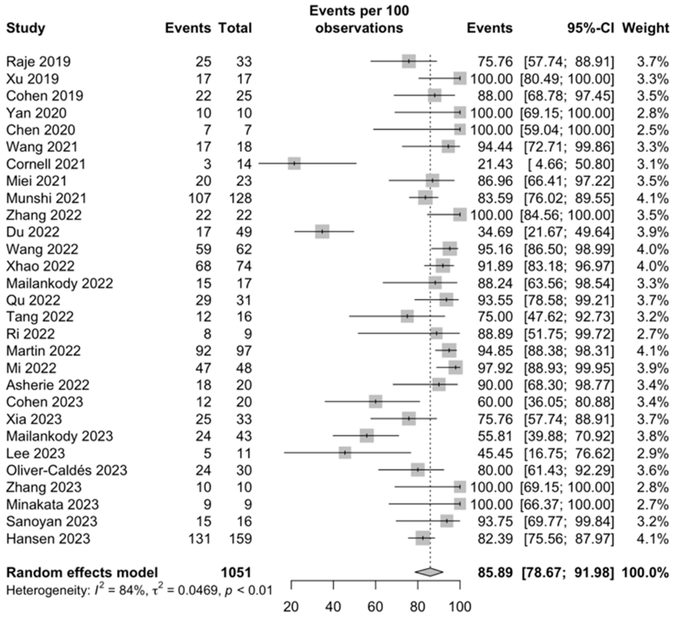
Study Events Total
Events per 100 observations
Events 95%-CI Weight
Raje 2019 25 33 75.76 [57.74; 88.91] 3.7%
Xu 2019 17 17 100.00 [80.49; 100.00] 3.3%
Cohen 2019 22 25 88.00 [68.78; 97.45] 3.5%
Yan 2020 10 10 100.00 [69.15; 100.00] 2.8%
Chen 2020 7 7 100.00 [59.04; 100.00] 2.5%
Wang 2021 17 18 94.44 [72.71; 99.86] 3.3%
Cornell 2021 3 14 21.43 [4.66; 50.80] 3.1%
Miei 2021 20 23 86.96 [66.41; 97.22] 3.5%
Munshi 2021 107 128 83.59 [76.02; 89.55] 4.1%
Zhang 2022 22 22 100.00 [84.56; 100.00] 3.5%
Du 2022 17 49 34.69 [21.67; 49.64] 3.9%
Wang 2022 59 62 95.16 [86.50; 98.99] 4.0%
Xhao 2022 68 74 91.89 [83.18; 96.97] 4.0%
Mailankody 2022 15 17 88.24 [63.56; 98.54] 3.3%
Qu 2022 29 31 93.55 [78.58; 99.21] 3.7%
Tang 2022 12 16 75.00 [47.62; 92.73] 3.2%
Ri 2022 8 9 88.89 [51.75; 99.72] 2.7%
Martin 2022 92 97 94.85 [88.38; 98.31] 4.1%
Mi 2022 47 48 97.92 [88.93; 99.95] 3.9%
Asherie 2022 18 20 90.00 [68.30; 98.77] 3.4%
Cohen 2023 12 20 60.00 [36.05; 80.88] 3.4%
Xia 2023 25 33 75.76 [57.74; 88.91] 3.7%
Mailankody 2023 24 43 55.81 [39.88; 70.92] 3.8%
Lee 2023 5 11 45.45 [16.75; 76.62] 2.9%
Oliver-Caldés 2023 24 30 80.00 [61.43; 92.29] 3.6%
Zhang 2023 10 10 100.00 [69.15; 100.00] 2.8%
Minakata 2023 9 9 100.00 [66.37; 100.00] 2.7%
Sanoyan 2023 15 16 93.75 [69.77; 99.84] 3.2%
Hansen 2023 131 159 82.39 [75.56; 87.97] 4.1%
Random effects model 1051 85.89 [78.67; 91.98] 100.0%
Heterogeneity: I^2 = 84%, τ^2 = 0.0469, p < 0.01
20 40 60 80 100

(A)

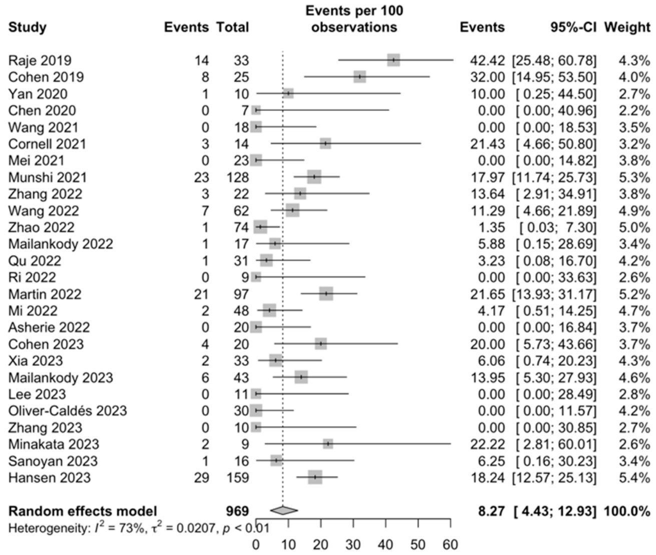
Study Events Total
Events per 100 observations
Events 95%-CI Weight
Raje 2019 14 33 42.42 [25.48; 60.78] 4.3%
Cohen 2019 8 25 32.00 [14.95; 53.50] 4.0%
Yan 2020 1 10 10.00 [0.25; 44.50] 2.7%
Chen 2020 0 7 0.00 [0.00; 40.96] 2.2%
Wang 2021 0 18 0.00 [0.00; 18.53] 3.5%
Cornell 2021 3 14 21.43 [4.66; 50.80] 3.2%
Mei 2021 0 23 0.00 [0.00; 14.82] 3.8%
Munshi 2021 23 128 17.97 [11.74; 25.73] 5.3%
Zhang 2022 3 22 13.64 [2.91; 34.91] 3.8%
Wang 2022 7 62 11.29 [4.66; 21.89] 4.9%
Zhao 2022 1 74 1.35 [0.03; 7.30] 5.0%
Mailankody 2022 1 17 5.88 [0.15; 28.69] 3.4%
Qu 2022 1 31 3.23 [0.08; 16.70] 4.2%
Ri 2022 0 9 0.00 [0.00; 33.63] 2.6%
Martin 2022 21 97 21.65 [13.93; 31.17] 5.2%
Mi 2022 2 48 4.17 [0.51; 14.25] 4.7%
Asherie 2022 0 20 0.00 [0.00; 16.84] 3.7%
Cohen 2023 4 20 20.00 [5.73; 43.66] 3.7%
Xia 2023 2 33 6.06 [0.74; 20.23] 4.3%
Mailankody 2023 6 43 13.95 [5.30; 27.93] 4.6%
Lee 2023 0 11 0.00 [0.00; 28.49] 2.8%
Oliver-Caldés 2023 0 30 0.00 [0.00; 11.57] 4.2%
Zhang 2023 0 10 0.00 [0.00; 30.85] 2.7%
Minakata 2023 2 9 22.22 [2.81; 60.01] 2.6%
Sanoyan 2023 1 16 6.25 [0.16; 30.23] 3.4%
Hansen 2023 29 159 18.24 [12.57; 25.13] 5.4%
Random effects model 969 8.27 [4.43; 12.93] 100.0%
Heterogeneity: I^2 = 73%, τ^2 = 0.0207, p < 0.01
0 10 20 30 40 50 60

(B)

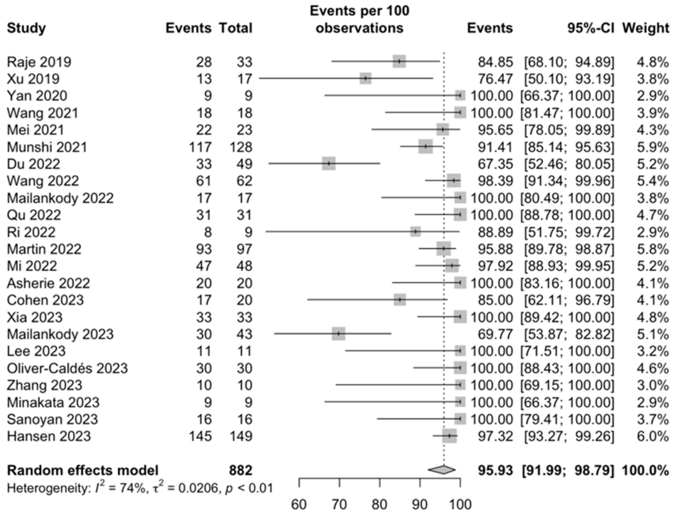
Study Events Total
Events per 100 observations
Events 95%-CI Weight
Raje 2019 28 33 84.85 [68.10; 94.89] 4.8%
Xu 2019 13 17 76.47 [50.10; 93.19] 3.8%
Yan 2020 9 9 100.00 [66.37; 100.00] 2.9%
Wang 2021 18 18 100.00 [81.47; 100.00] 3.9%
Mei 2021 22 23 95.65 [78.05; 99.89] 4.3%
Munshi 2021 117 128 91.41 [85.14; 95.63] 5.9%
Du 2022 33 49 67.35 [52.46; 80.05] 5.2%
Wang 2022 61 62 98.39 [91.34; 99.96] 5.4%
Mailankody 2022 17 17 100.00 [80.49; 100.00] 3.8%
Qu 2022 31 31 100.00 [88.78; 100.00] 4.7%
Ri 2022 8 9 88.89 [51.75; 99.72] 2.9%
Martin 2022 93 97 95.88 [89.78; 98.87] 5.8%
Mi 2022 47 48 97.92 [88.93; 99.95] 5.2%
Asherie 2022 20 20 100.00 [83.16; 100.00] 4.1%
Cohen 2023 17 20 85.00 [62.11; 96.79] 4.1%
Xia 2023 33 33 100.00 [89.42; 100.00] 4.8%
Mailankody 2023 30 43 69.77 [53.87; 82.82] 5.1%
Lee 2023 11 11 100.00 [71.51; 100.00] 3.2%
Oliver-Caldés 2023 30 30 100.00 [88.43; 100.00] 4.6%
Zhang 2023 10 10 100.00 [69.15; 100.00] 3.0%
Minakata 2023 9 9 100.00 [66.37; 100.00] 2.9%
Sanoyan 2023 16 16 100.00 [79.41; 100.00] 3.7%
Hansen 2023 145 149 97.32 [93.27; 99.26] 6.0%
Random effects model 882 95.93 [91.99; 98.79] 100.0%
Heterogeneity: I^2 = 74%, τ^2 = 0.0206, p < 0.01
60 70 80 90 100

(C)

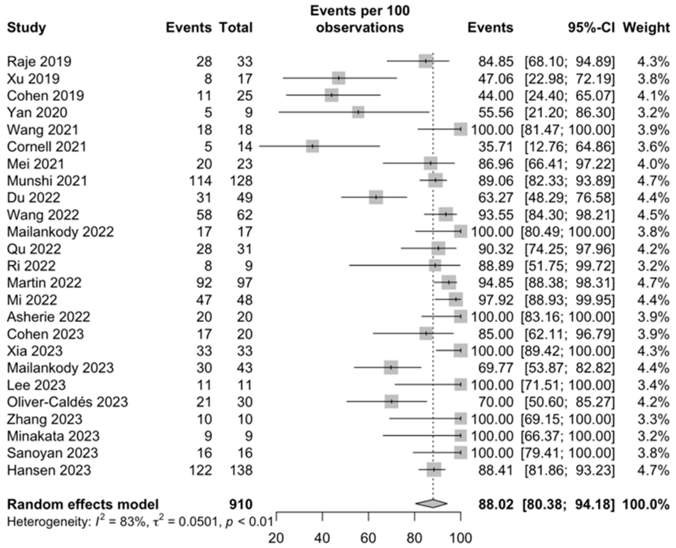
Study Events Total
Events per 100 observations
Events 95%-CI Weight
Raje 2019 28 33 84.85 [68.10; 94.89] 4.3%
Xu 2019 8 17 47.06 [22.98; 72.19] 3.8%
Cohen 2019 11 25 44.00 [24.40; 65.07] 4.1%
Yan 2020 5 9 55.56 [21.20; 86.30] 3.2%
Wang 2021 18 18 100.00 [81.47; 100.00] 3.9%
Cornell 2021 5 14 35.71 [12.76; 64.86] 3.6%
Mei 2021 20 23 86.96 [66.41; 97.22] 4.0%
Munshi 2021 114 128 89.06 [82.33; 93.89] 4.7%
Du 2022 31 49 63.27 [48.29; 76.58] 4.4%
Wang 2022 58 62 93.55 [84.30; 98.21] 4.5%
Mailankody 2022 17 17 100.00 [80.49; 100.00] 3.8%
Qu 2022 28 31 90.32 [74.25; 97.96] 4.2%
Ri 2022 8 9 88.89 [51.75; 99.72] 3.2%
Martin 2022 92 97 94.85 [88.38; 98.31] 4.7%
Mi 2022 47 48 97.92 [88.93; 99.95] 4.4%
Asherie 2022 20 20 100.00 [83.16; 100.00] 3.9%
Cohen 2023 17 20 85.00 [62.11; 96.79] 3.9%
Xia 2023 33 33 100.00 [89.42; 100.00] 4.3%
Mailankody 2023 30 43 69.77 [53.87; 82.82] 4.4%
Lee 2023 11 11 100.00 [71.51; 100.00] 3.4%
Oliver-Caldés 2023 21 30 70.00 [50.60; 85.27] 4.2%
Zhang 2023 10 10 100.00 [69.15; 100.00] 3.3%
Minakata 2023 9 9 100.00 [66.37; 100.00] 3.2%
Sanoyan 2023 16 16 100.00 [79.41; 100.00] 3.8%
Hansen 2023 122 138 88.41 [81.86; 93.23] 4.7%
Random effects model 910 88.02 [80.38; 94.18] 100.0%
Heterogeneity: I^2 = 83%, τ^2 = 0.0501, p < 0.01
20 40 60 80 100

(D)

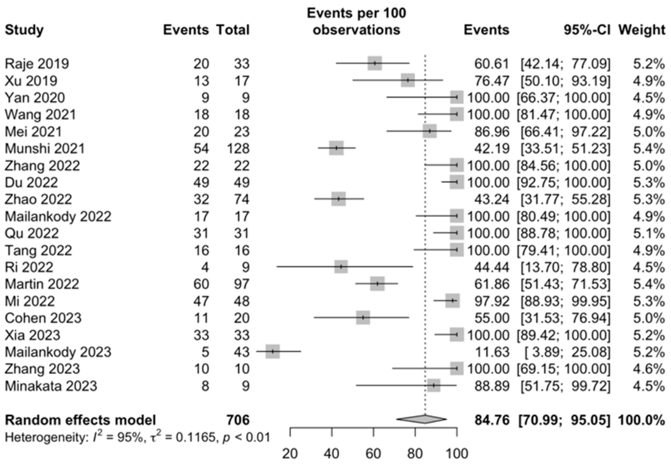
Study Events Total
Events per 100 observations
Events 95%-CI Weight
Raje 2019 20 33 60.61 [42.14; 77.09] 5.2%
Xu 2019 13 17 76.47 [50.10; 93.19] 4.9%
Yan 2020 9 9 100.00 [66.37; 100.00] 4.5%
Wang 2021 18 18 100.00 [81.47; 100.00] 4.9%
Mei 2021 20 23 86.96 [66.41; 97.22] 5.0%
Munshi 2021 54 128 42.19 [33.51; 51.23] 5.4%
Zhang 2022 22 22 100.00 [84.56; 100.00] 5.0%
Du 2022 49 49 100.00 [92.75; 100.00] 5.3%
Zhao 2022 32 74 43.24 [31.77; 55.28] 5.3%
Mailankody 2022 17 17 100.00 [80.49; 100.00] 4.9%
Qu 2022 31 31 100.00 [88.78; 100.00] 5.1%
Tang 2022 16 16 100.00 [79.41; 100.00] 4.9%
Ri 2022 4 9 44.44 [13.70; 78.80] 4.5%
Martin 2022 60 97 61.86 [51.43; 71.53] 5.4%
Mi 2022 47 48 97.92 [88.93; 99.95] 5.3%
Cohen 2023 11 20 55.00 [31.53; 76.94] 5.0%
Xia 2023 33 33 100.00 [89.42; 100.00] 5.2%
Mailankody 2023 5 43 11.63 [3.89; 25.08] 5.2%
Zhang 2023 10 10 100.00 [69.15; 100.00] 4.6%
Minakata 2023 8 9 88.89 [51.75; 99.72] 4.5%
Random effects model 706 84.76 [70.99; 95.05] 100.0%
Heterogeneity: I^2 = 95%, τ^2 = 0.1165, p < 0.01
20 40 60 80 100

(E)

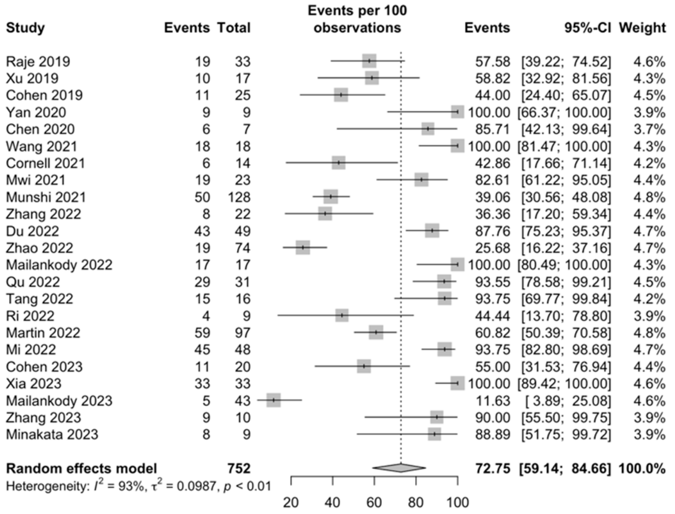
Study Events Total
Events per 100 observations
Events 95%-CI Weight
Raje 2019 19 33 57.58 [39.22; 74.52] 4.6%
Xu 2019 10 17 58.82 [32.92; 81.56] 4.3%
Cohen 2019 11 25 44.00 [24.40; 65.07] 4.5%
Yan 2020 9 9 100.00 [66.37; 100.00] 3.9%
Chen 2020 6 7 85.71 [42.13; 99.64] 3.7%
Wang 2021 18 18 100.00 [81.47; 100.00] 4.3%
Cornell 2021 6 14 42.86 [17.66; 71.14] 4.2%
Mwi 2021 19 23 82.61 [61.22; 95.05] 4.4%
Munshi 2021 50 128 39.06 [30.56; 48.08] 4.8%
Zhang 2022 8 22 36.36 [17.20; 59.34] 4.4%
Du 2022 43 49 87.76 [75.23; 95.37] 4.7%
Zhao 2022 19 74 25.68 [16.22; 37.16] 4.7%
Mailankody 2022 17 17 100.00 [80.49; 100.00] 4.3%
Qu 2022 29 31 93.55 [78.58; 99.21] 4.5%
Tang 2022 15 16 93.75 [69.77; 99.84] 4.2%
Ri 2022 4 9 44.44 [13.70; 78.80] 3.9%
Martin 2022 59 97 60.82 [50.39; 70.58] 4.8%
Mi 2022 45 48 93.75 [82.80; 98.69] 4.7%
Cohen 2023 11 20 55.00 [31.53; 76.94] 4.4%
Xia 2023 33 33 100.00 [89.42; 100.00] 4.6%
Mailankody 2023 5 43 11.63 [3.89; 25.08] 4.6%
Zhang 2023 9 10 90.00 [55.50; 99.75] 3.9%
Minakata 2023 8 9 88.89 [51.75; 99.72] 3.9%
Random effects model 752 72.75 [59.14; 84.66] 100.0%
Heterogeneity: I^2 = 93%, τ^2 = 0.0987, p < 0.01
20 40 60 80 100

(F)

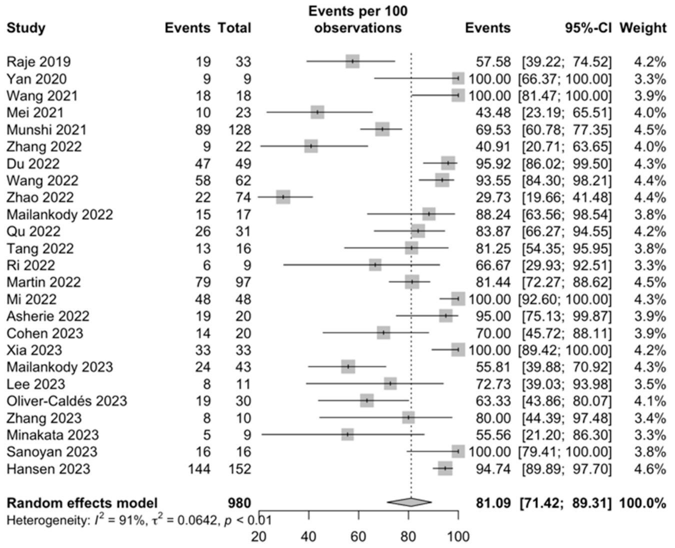
Study Events Total
Events per 100 observations
Events 95%-CI Weight
Raje 2019 19 33 57.58 [39.22; 74.52] 4.2%
Yan 2020 9 9 100.00 [66.37; 100.00] 3.3%
Wang 2021 18 18 100.00 [81.47; 100.00] 3.9%
Mei 2021 10 23 43.48 [23.19; 65.51] 4.0%
Munshi 2021 89 128 69.53 [60.78; 77.35] 4.5%
Zhang 2022 9 22 40.91 [20.71; 63.65] 4.0%
Du 2022 47 49 95.92 [86.02; 99.50] 4.3%
Wang 2022 58 62 93.55 [84.30; 98.21] 4.4%
Zhao 2022 22 74 29.73 [19.66; 41.48] 4.4%
Mailankody 2022 15 17 88.24 [63.56; 98.54] 3.8%
Qu 2022 26 31 83.87 [66.27; 94.55] 4.2%
Tang 2022 13 16 81.25 [54.35; 95.95] 3.8%
Ri 2022 6 9 66.67 [29.93; 92.51] 3.3%
Martin 2022 79 97 81.44 [72.27; 88.62] 4.5%
Mi 2022 48 48 100.00 [92.60; 100.00] 4.3%
Asherie 2022 19 20 95.00 [75.13; 99.87] 3.9%
Cohen 2023 14 20 70.00 [45.72; 88.11] 3.9%
Xia 2023 33 33 100.00 [89.42; 100.00] 4.2%
Mailankody 2023 24 43 55.81 [39.88; 70.92] 4.3%
Lee 2023 8 11 72.73 [39.03; 93.98] 3.5%
Oliver-Caldés 2023 19 30 63.33 [43.86; 80.07] 4.1%
Zhang 2023 8 10 80.00 [44.39; 97.48] 3.4%
Minakata 2023 5 9 55.56 [21.20; 86.30] 3.3%
Sanoyan 2023 16 16 100.00 [79.41; 100.00] 3.8%
Hansen 2023 144 152 94.74 [89.89; 97.70] 4.6%
Random effects model 980 81.09 [71.42; 89.31] 100.0%
Heterogeneity: I^2 = 91%, τ^2 = 0.0642, p < 0.01
20 40 60 80 100

(G)

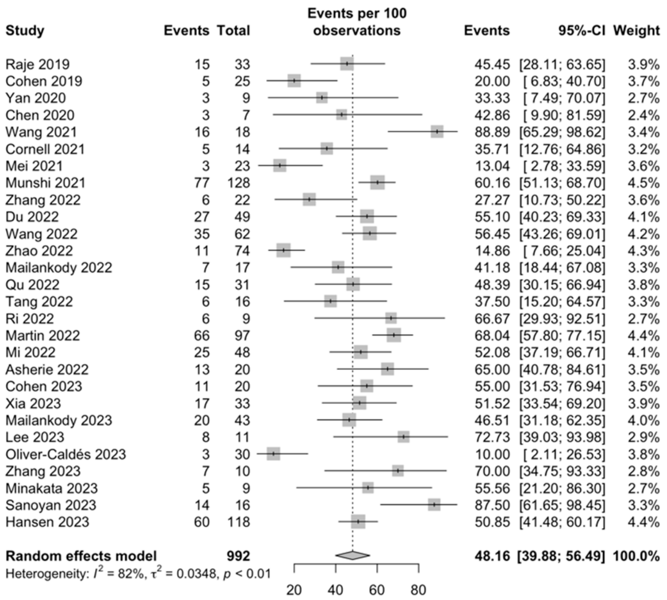
Study Events Total
Events per 100 observations
Events 95%-CI Weight
Raje 2019 15 33 45.45 [28.11; 63.65] 3.9%
Cohen 2019 5 25 20.00 [6.83; 40.70] 3.7%
Yan 2020 3 9 33.33 [7.49; 70.07] 2.7%
Chen 2020 3 7 42.86 [9.90; 81.59] 2.4%
Wang 2021 16 18 88.89 [65.29; 98.62] 3.4%
Cornell 2021 5 14 35.71 [12.76; 64.86] 3.2%
Mei 2021 3 23 13.04 [2.78; 33.59] 3.6%
Munshi 2021 77 128 60.16 [51.13; 68.70] 4.5%
Zhang 2022 6 22 27.27 [10.73; 50.22] 3.6%
Du 2022 27 49 55.10 [40.23; 69.33] 4.1%
Wang 2022 35 62 56.45 [43.26; 69.01] 4.2%
Zhao 2022 11 74 14.86 [7.66; 25.04] 4.3%
Mailankody 2022 7 17 41.18 [18.44; 67.08] 3.3%
Qu 2022 15 31 48.39 [30.15; 66.94] 3.8%
Tang 2022 6 16 37.50 [15.20; 64.57] 3.3%
Ri 2022 6 9 66.67 [29.93; 92.51] 2.7%
Martin 2022 66 97 68.04 [57.80; 77.15] 4.4%
Mi 2022 25 48 52.08 [37.19; 66.71] 4.1%
Asherie 2022 13 20 65.00 [40.78; 84.61] 3.5%
Cohen 2023 11 20 55.00 [31.53; 76.94] 3.5%
Xia 2023 17 33 51.52 [33.54; 69.20] 3.9%
Mailankody 2023 20 43 46.51 [31.18; 62.35] 4.0%
Lee 2023 8 11 72.73 [39.03; 93.98] 2.9%
Oliver-Caldés 2023 3 30 10.00 [2.11; 26.53] 3.8%
Zhang 2023 7 10 70.00 [34.75; 93.33] 2.8%
Minakata 2023 5 9 55.56 [21.20; 86.30] 2.7%
Sanoyan 2023 14 16 87.50 [61.65; 98.45] 3.3%
Hansen 2023 60 118 50.85 [41.48; 60.17] 4.4%
Random effects model 992 48.16 [39.88; 56.49] 100.0%
Heterogeneity: I^2 = 82%, τ^2 = 0.0348, p < 0.01
20 40 60 80

(H)

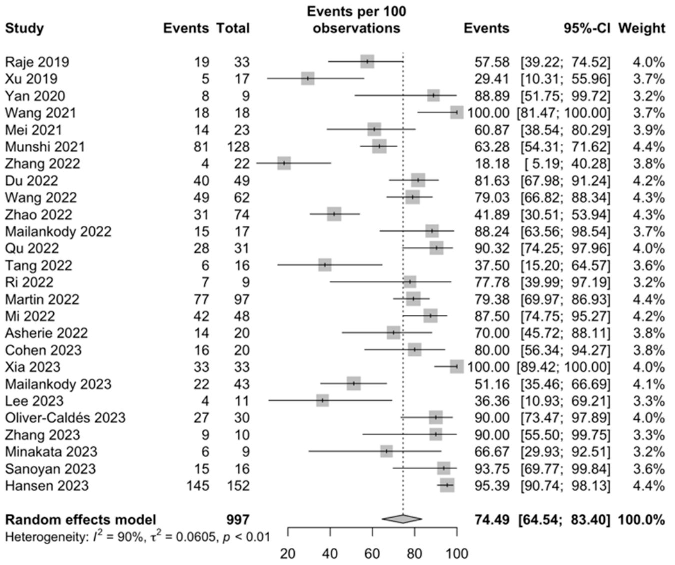
Study Events Total
Events per 100 observations
Events 95%-CI Weight
Raje 2019 19 33 57.58 [39.22; 74.52] 4.0%
Xu 2019 5 17 29.41 [10.31; 55.96] 3.7%
Yan 2020 8 9 88.89 [51.75; 99.72] 3.2%
Wang 2021 18 18 100.00 [81.47; 100.00] 3.7%
Mei 2021 14 23 60.87 [38.54; 80.29] 3.9%
Munshi 2021 81 128 63.28 [54.31; 71.62] 4.4%
Zhang 2022 4 22 18.18 [5.19; 40.28] 3.8%
Du 2022 40 49 81.63 [67.98; 91.24] 4.2%
Wang 2022 49 62 79.03 [66.82; 88.34] 4.3%
Zhao 2022 31 74 41.89 [30.51; 53.94] 4.3%
Mailankody 2022 15 17 88.24 [63.56; 98.54] 3.7%
Qu 2022 28 31 90.32 [74.25; 97.96] 4.0%
Tang 2022 6 16 37.50 [15.20; 64.57] 3.6%
Ri 2022 7 9 77.78 [39.99; 97.19] 3.2%
Martin 2022 77 97 79.38 [69.97; 86.93] 4.4%
Mi 2022 42 48 87.50 [74.75; 95.27] 4.2%
Asherie 2022 14 20 70.00 [45.72; 88.11] 3.8%
Cohen 2023 16 20 80.00 [56.34; 94.27] 3.8%
Xia 2023 33 33 100.00 [89.42; 100.00] 4.0%
Mailankody 2023 22 43 51.16 [35.46; 66.69] 4.1%
Lee 2023 4 11 36.36 [10.93; 69.21] 3.3%
Oliver-Caldés 2023 27 30 90.00 [73.47; 97.89] 4.0%
Zhang 2023 9 10 90.00 [55.50; 99.75] 3.3%
Minakata 2023 6 9 66.67 [29.93; 92.51] 3.2%
Sanoyan 2023 15 16 93.75 [69.77; 99.84] 3.6%
Hansen 2023 145 152 95.39 [90.74; 98.13] 4.4%
Random effects model 997 74.49 [64.54; 83.40] 100.0%
Heterogeneity: I^2 = 90%, τ^2 = 0.0605, p < 0.01
20 40 60 80 100

(I)

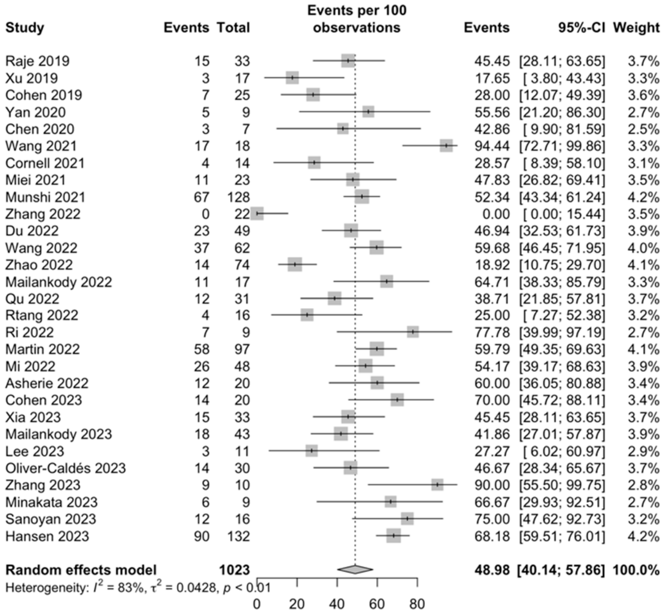
Study Events Total
Events per 100 observations
Events 95%-CI Weight
Raje 2019 15 33 45.45 [28.11; 63.65] 3.7%
Xu 2019 3 17 17.65 [3.80; 43.43] 3.3%
Cohen 2019 7 25 28.00 [12.07; 49.39] 3.6%
Yan 2020 5 9 55.56 [21.20; 86.30] 2.7%
Chen 2020 3 7 42.86 [9.90; 81.59] 2.5%
Wang 2021 17 18 94.44 [72.71; 99.86] 3.3%
Cornell 2021 4 14 28.57 [8.39; 58.10] 3.1%
Miei 2021 11 23 47.83 [26.82; 69.41] 3.5%
Munshi 2021 67 128 52.34 [43.34; 61.24] 4.2%
Zhang 2022 0 22 0.00 [0.00; 15.44] 3.5%
Du 2022 23 49 46.94 [32.53; 61.73] 3.9%
Wang 2022 37 62 59.68 [46.45; 71.95] 4.0%
Zhao 2022 14 74 18.92 [10.75; 29.70] 4.1%
Mailankody 2022 11 17 64.71 [38.33; 85.79] 3.3%
Qu 2022 12 31 38.71 [21.85; 57.81] 3.7%
Rtang 2022 4 16 25.00 [7.27; 52.38] 3.2%
Ri 2022 7 9 77.78 [39.99; 97.19] 2.7%
Martin 2022 58 97 59.79 [49.35; 69.63] 4.1%
Mi 2022 26 48 54.17 [39.17; 68.63] 3.9%
Asherie 2022 12 20 60.00 [36.05; 80.88] 3.4%
Cohen 2023 14 20 70.00 [45.72; 88.11] 3.4%
Xia 2023 15 33 45.45 [28.11; 63.65] 3.7%
Mailankody 2023 18 43 41.86 [27.01; 57.87] 3.9%
Lee 2023 3 11 27.27 [6.02; 60.97] 2.9%
Oliver-Caldés 2023 14 30 46.67 [28.34; 65.67] 3.7%
Zhang 2023 9 10 90.00 [55.50; 99.75] 2.8%
Minakata 2023 6 9 66.67 [29.93; 92.51] 2.7%
Sanoyan 2023 12 16 75.00 [47.62; 92.73] 3.2%
Hansen 2023 90 132 68.18 [59.51; 76.01] 4.2%
Random effects model 1023 48.98 [40.14; 57.86] 100.0%
Heterogeneity: I^2 = 83%, τ^2 = 0.0428, p < 0.01
0 20 40 60 80

(J)

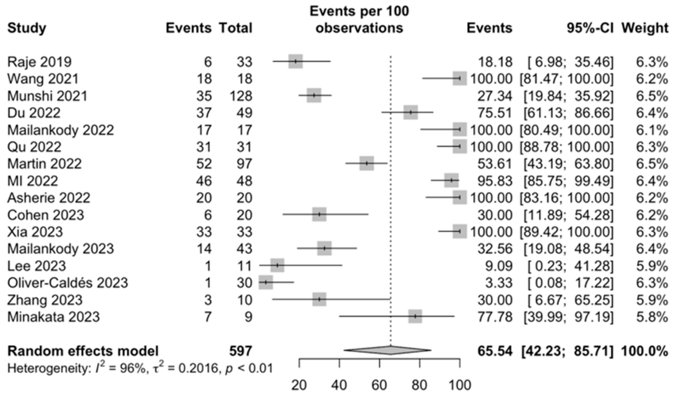
Study Events Total
Events per 100 observations
Events 95%-CI Weight
Raje 2019 6 33 18.18 [6.98; 35.46] 6.3%
Wang 2021 18 18 100.00 [81.47; 100.00] 6.2%
Munshi 2021 35 128 27.34 [19.84; 35.92] 6.5%
Du 2022 37 49 75.51 [61.13; 86.66] 6.4%
Mailankody 2022 17 17 100.00 [80.49; 100.00] 6.1%
Qu 2022 31 31 100.00 [88.78; 100.00] 6.3%
Martin 2022 52 97 53.61 [43.19; 63.80] 6.5%
MI 2022 46 48 95.83 [85.75; 99.49] 6.4%
Asherie 2022 20 20 100.00 [83.16; 100.00] 6.2%
Cohen 2023 6 20 30.00 [11.89; 54.28] 6.2%
Xia 2023 33 33 100.00 [89.42; 100.00] 6.3%
Mailankody 2023 14 43 32.56 [19.08; 48.54] 6.4%
Lee 2023 1 11 9.09 [0.23; 41.28] 5.9%
Oliver-Caldés 2023 1 30 3.33 [0.08; 17.22] 6.3%
Zhang 2023 3 10 30.00 [6.67; 65.25] 5.9%
Minakata 2023 7 9 77.78 [39.99; 97.19] 5.8%
Random effects model 597 65.54 [42.23; 85.71] 100.0%
Heterogeneity: I^2 = 96%, τ^2 = 0.2016, p < 0.01
20 40 60 80 100

(K)

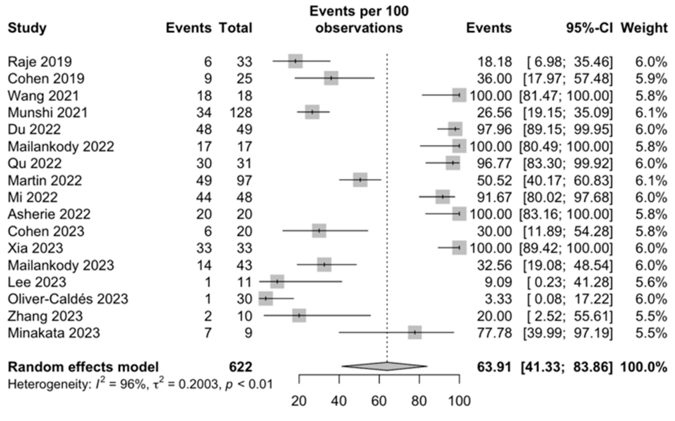
Study Events Total
Events per 100 observations
Events 95%-CI Weight
Raje 2019 6 33 18.18 [6.98; 35.46] 6.0%
Cohen 2019 9 25 36.00 [17.97; 57.48] 5.9%
Wang 2021 18 18 100.00 [81.47; 100.00] 5.8%
Munshi 2021 34 128 26.56 [19.15; 35.09] 6.1%
Du 2022 48 49 97.96 [89.15; 99.95] 6.0%
Mailankody 2022 17 17 100.00 [80.49; 100.00] 5.8%
Qu 2022 30 31 96.77 [83.30; 99.92] 6.0%
Martin 2022 49 97 50.52 [40.17; 60.83] 6.1%
Mi 2022 44 48 91.67 [80.02; 97.68] 6.0%
Asherie 2022 20 20 100.00 [83.16; 100.00] 5.8%
Cohen 2023 6 20 30.00 [11.89; 54.28] 5.8%
Xia 2023 33 33 100.00 [89.42; 100.00] 6.0%
Mailankody 2023 14 43 32.56 [19.08; 48.54] 6.0%
Lee 2023 1 11 9.09 [0.23; 41.28] 5.6%
Oliver-Caldés 2023 1 30 3.33 [0.08; 17.22] 6.0%
Zhang 2023 2 10 20.00 [2.52; 55.61] 5.5%
Minakata 2023 7 9 77.78 [39.99; 97.19] 5.5%
Random effects model 622 63.91 [41.33; 83.86] 100.0%
Heterogeneity: I^2 = 96%, τ^2 = 0.2003, p < 0.01
20 40 60 80 100

(L)

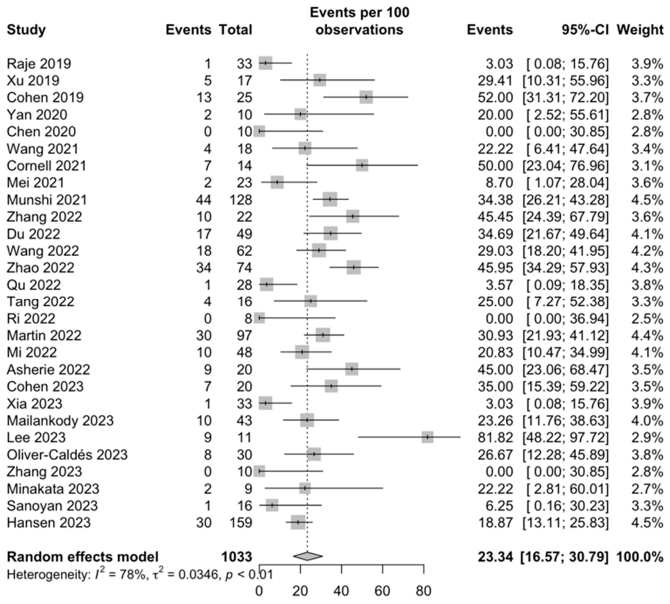

(M)

Figure S2. Safety outcomes: **(A)** any grade CRS; **(B)** any grade neurotoxicity; **(C)** any grade neutropenia; **(D)** grade ≥ 3 neutropenia; **(E)** any grade leukopenia; **(F)** grade ≥ 3 leukopenia; **(G)** any grade anemia; **(H)** grade ≥ 3 anemia; **(I)** any grade thrombocytopenia; **(J)** grade ≥ 3 thrombocytopenia **(K)** any grade lymphopenia; **(L)** grade ≥ 3 lymphopenia; (**M)** all-cause mortality.

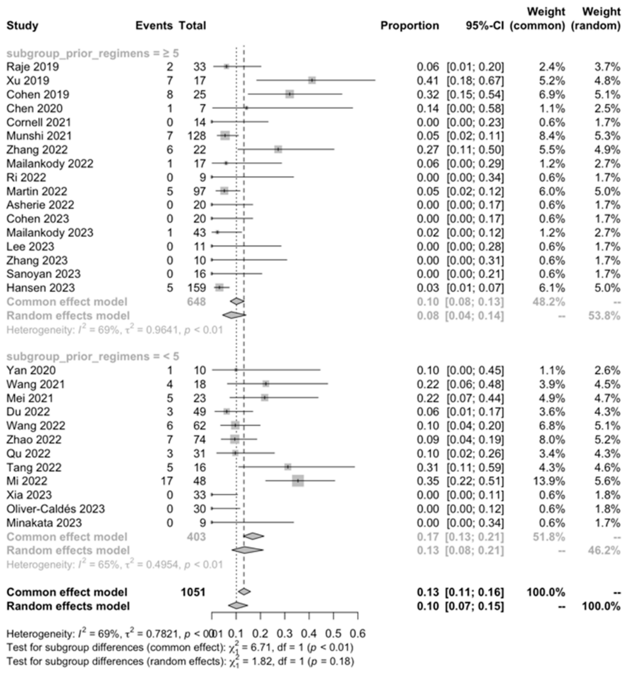
Study Events Total Proportion 95%-CI Weight (common) Weight (random)
subgroup_prior_regimens = ≥ 5
Raje 2019 2 33 0.06 [0.01; 0.20] 2.4% 3.7%
Xu 2019 7 17 0.41 [0.18; 0.67] 5.2% 4.8%
Cohen 2019 8 25 0.32 [0.15; 0.54] 6.9% 5.1%
Chen 2020 1 7 0.14 [0.00; 0.58] 1.1% 2.5%
Cornell 2021 0 14 0.00 [0.00; 0.23] 0.6% 1.7%
Munshi 2021 7 128 0.05 [0.02; 0.11] 8.4% 5.3%
Zhang 2022 6 22 0.27 [0.11; 0.50] 5.5% 4.9%
Mailankody 2022 1 17 0.06 [0.00; 0.29] 1.2% 2.7%
Ri 2022 0 9 0.00 [0.00; 0.34] 0.6% 1.7%
Martin 2022 5 97 0.05 [0.02; 0.12] 6.0% 5.0%
Asherie 2022 0 20 0.00 [0.00; 0.17] 0.6% 1.7%
Cohen 2023 0 20 0.00 [0.00; 0.17] 0.6% 1.7%
Mailankody 2023 1 43 0.02 [0.00; 0.12] 1.2% 2.7%
Lee 2023 0 11 0.00 [0.00; 0.28] 0.6% 1.7%
Zhang 2023 0 10 0.00 [0.00; 0.31] 0.6% 1.7%
Sanoyan 2023 0 16 0.00 [0.00; 0.21] 0.6% 1.7%
Hansen 2023 5 159 0.03 [0.01; 0.07] 6.1% 5.0%
Common effect model 648 0.10 [0.08; 0.13] 48.2% --
Random effects model 0.08 [0.04; 0.14] -- 53.8%
Heterogeneity: I^2 = 69%, τ^2 = 0.9641, p < 0.01
subgroup_prior_regimens = < 5
Yan 2020 1 10 0.10 [0.00; 0.45] 1.1% 2.6%
Wang 2021 4 18 0.22 [0.06; 0.48] 3.9% 4.5%
Mei 2021 5 23 0.22 [0.07; 0.44] 4.9% 4.7%
Du 2022 3 49 0.06 [0.01; 0.17] 3.6% 4.3%
Wang 2022 6 62 0.10 [0.04; 0.20] 6.8% 5.1%
Zhao 2022 7 74 0.09 [0.04; 0.19] 8.0% 5.2%
Qu 2022 3 31 0.10 [0.02; 0.26] 3.4% 4.3%
Tang 2022 5 16 0.31 [0.11; 0.59] 4.3% 4.6%
Mi 2022 17 48 0.35 [0.22; 0.51] 13.9% 5.6%
Xia 2023 0 33 0.00 [0.00; 0.11] 0.6% 1.8%
Oliver-Caldés 2023 0 30 0.00 [0.00; 0.12] 0.6% 1.8%
Minakata 2023 0 9 0.00 [0.00; 0.34] 0.6% 1.7%
Common effect model 403 0.17 [0.13; 0.21] 51.8% --
Random effects model 0.13 [0.08; 0.21] -- 46.2%
Heterogeneity: I^2 = 65%, τ^2 = 0.4954, p < 0.01
Common effect model 1051 0.13 [0.11; 0.16] 100.0% --
Random effects model 0.10 [0.07; 0.15] -- 100.0%
Heterogeneity: I^2 = 69%, τ^2 = 0.7821, p < 0 0.1 0.1 0.2 0.3 0.4 0.5 0.6
Test for subgroup differences (common effect): χ^2_1 = 6.71, df = 1 (p < 0.01)
Test for subgroup differences (random effects): χ^2_1 = 1.82, df = 1 (p = 0.18)

(A)

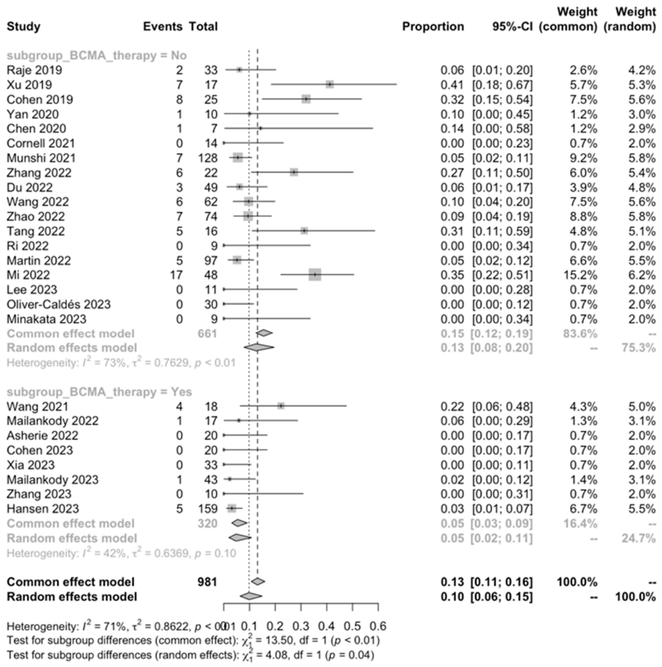
Study Events Total Proportion 95%-CI Weight (common) Weight (random)
subgroup_BCMA_therapy = No
Raje 2019 2 33 0.06 [0.01; 0.20] 2.6% 4.2%
Xu 2019 7 17 0.41 [0.18; 0.67] 5.7% 5.3%
Cohen 2019 8 25 0.32 [0.15; 0.54] 7.5% 5.6%
Yan 2020 1 10 0.10 [0.00; 0.45] 1.2% 3.0%
Chen 2020 1 7 0.14 [0.00; 0.58] 1.2% 2.9%
Cornell 2021 0 14 0.00 [0.00; 0.23] 0.7% 2.0%
Munshi 2021 7 128 0.05 [0.02; 0.11] 9.2% 5.8%
Zhang 2022 6 22 0.27 [0.11; 0.50] 6.0% 5.4%
Du 2022 3 49 0.06 [0.01; 0.17] 3.9% 4.8%
Wang 2022 6 62 0.10 [0.04; 0.20] 7.5% 5.6%
Zhao 2022 7 74 0.09 [0.04; 0.19] 8.8% 5.8%
Tang 2022 5 16 0.31 [0.11; 0.59] 4.8% 5.1%
Ri 2022 0 9 0.00 [0.00; 0.34] 0.7% 2.0%
Martin 2022 5 97 0.05 [0.02; 0.12] 6.6% 5.5%
Mi 2022 17 48 0.35 [0.22; 0.51] 15.2% 6.2%
Lee 2023 0 11 0.00 [0.00; 0.28] 0.7% 2.0%
Oliver-Caldés 2023 0 30 0.00 [0.00; 0.12] 0.7% 2.0%
Minakata 2023 0 9 0.00 [0.00; 0.34] 0.7% 2.0%
Common effect model 661 0.15 [0.12; 0.19] 83.6% --
Random effects model 0.13 [0.08; 0.20] -- 75.3%
Heterogeneity: I^2 = 73%, τ^2 = 0.7629, p < 0.01
subgroup_BCMA_therapy = Yes
Wang 2021 4 18 0.22 [0.06; 0.48] 4.3% 5.0%
Mailankody 2022 1 17 0.06 [0.00; 0.29] 1.3% 3.1%
Asherie 2022 0 20 0.00 [0.00; 0.17] 0.7% 2.0%
Cohen 2023 0 20 0.00 [0.00; 0.17] 0.7% 2.0%
Xia 2023 0 33 0.00 [0.00; 0.11] 0.7% 2.0%
Mailankody 2023 1 43 0.02 [0.00; 0.12] 1.4% 3.1%
Zhang 2023 0 10 0.00 [0.00; 0.31] 0.7% 2.0%
Hansen 2023 5 159 0.03 [0.01; 0.07] 6.7% 5.5%
Common effect model 320 0.05 [0.03; 0.09] 16.4% --
Random effects model 0.05 [0.02; 0.11] -- 24.7%
Heterogeneity: I^2 = 42%, τ^2 = 0.6369, p = 0.10
Common effect model 981 0.13 [0.11; 0.16] 100.0% --
Random effects model 0.10 [0.06; 0.15] -- 100.0%
Heterogeneity: I^2 = 71%, τ^2 = 0.8622, p < 0 0.1 0.1 0.2 0.3 0.4 0.5 0.6
Test for subgroup differences (common effect): χ^2_1 = 13.50, df = 1 (p < 0.01)
Test for subgroup differences (random effects): χ^2_1 = 4.08, df = 1 (p = 0.04)

(B)

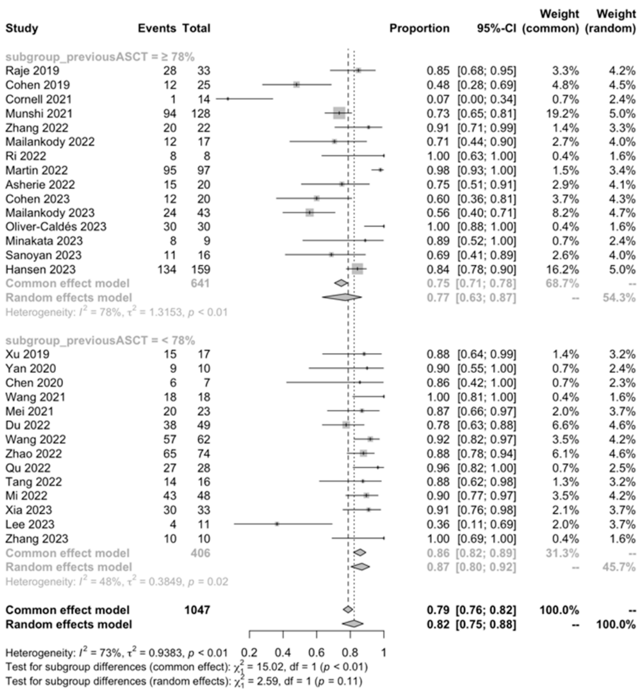
Study Events Total Proportion 95%-CI Weight (common) Weight (random)
subgroup_previousASCT = ≥ 78%
Raje 2019 28 33 0.85 [0.68; 0.95] 3.3% 4.2%
Cohen 2019 12 25 0.48 [0.28; 0.69] 4.8% 4.5%
Cornell 2021 1 14 0.07 [0.00; 0.34] 0.7% 2.4%
Munshi 2021 94 128 0.73 [0.65; 0.81] 19.2% 5.0%
Zhang 2022 20 22 0.91 [0.71; 0.99] 1.4% 3.3%
Mailankody 2022 12 17 0.71 [0.44; 0.90] 2.7% 4.0%
Ri 2022 8 8 1.00 [0.63; 1.00] 0.4% 1.6%
Martin 2022 95 97 0.98 [0.93; 1.00] 1.5% 3.4%
Asherie 2022 15 20 0.75 [0.51; 0.91] 2.9% 4.1%
Cohen 2023 12 20 0.60 [0.36; 0.81] 3.7% 4.3%
Mailankody 2023 24 43 0.56 [0.40; 0.71] 8.2% 4.7%
Oliver-Caldés 2023 30 30 1.00 [0.88; 1.00] 0.4% 1.6%
Minakata 2023 8 9 0.89 [0.52; 1.00] 0.7% 2.4%
Sanoyan 2023 11 16 0.69 [0.41; 0.89] 2.6% 4.0%
Hansen 2023 134 159 0.84 [0.78; 0.90] 16.2% 5.0%
Common effect model 641 0.75 [0.71; 0.78] 68.7% --
Random effects model 0.77 [0.63; 0.87] -- 54.3%
Heterogeneity: I^2 = 78%, τ^2 = 1.3153, p < 0.01
subgroup_previousASCT = < 78%
Xu 2019 15 17 0.88 [0.64; 0.99] 1.4% 3.2%
Yan 2020 9 10 0.90 [0.55; 1.00] 0.7% 2.4%
Chen 2020 6 7 0.86 [0.42; 1.00] 0.7% 2.3%
Wang 2021 18 18 1.00 [0.81; 1.00] 0.4% 1.6%
Mei 2021 20 23 0.87 [0.66; 0.97] 2.0% 3.7%
Du 2022 38 49 0.78 [0.63; 0.88] 6.6% 4.6%
Wang 2022 57 62 0.92 [0.82; 0.97] 3.5% 4.2%
Zhao 2022 65 74 0.88 [0.78; 0.94] 6.1% 4.6%
Qu 2022 27 28 0.96 [0.82; 1.00] 0.7% 2.5%
Tang 2022 14 16 0.88 [0.62; 0.98] 1.3% 3.2%
Mi 2022 43 48 0.90 [0.77; 0.97] 3.5% 4.2%
Xia 2023 30 33 0.91 [0.76; 0.98] 2.1% 3.7%
Lee 2023 4 11 0.36 [0.11; 0.69] 2.0% 3.7%
Zhang 2023 10 10 1.00 [0.69; 1.00] 0.4% 1.6%
Common effect model 406 0.86 [0.82; 0.89] 31.3% --
Random effects model 0.87 [0.80; 0.92] -- 45.7%
Heterogeneity: I^2 = 48%, τ^2 = 0.3849, p = 0.02
Common effect model 1047 0.79 [0.76; 0.82] 100.0% --
Random effects model 0.82 [0.75; 0.88] -- 100.0%
Heterogeneity: I^2 = 73%, τ^2 = 0.9383, p < 0.01 0.2 0.4 0.6 0.8 1
Test for subgroup differences (common effect): χ^2_1 = 15.02, df = 1 (p < 0.01)
Test for subgroup differences (random effects): χ^2_1 = 2.59, df = 1 (p = 0.11)

(C)

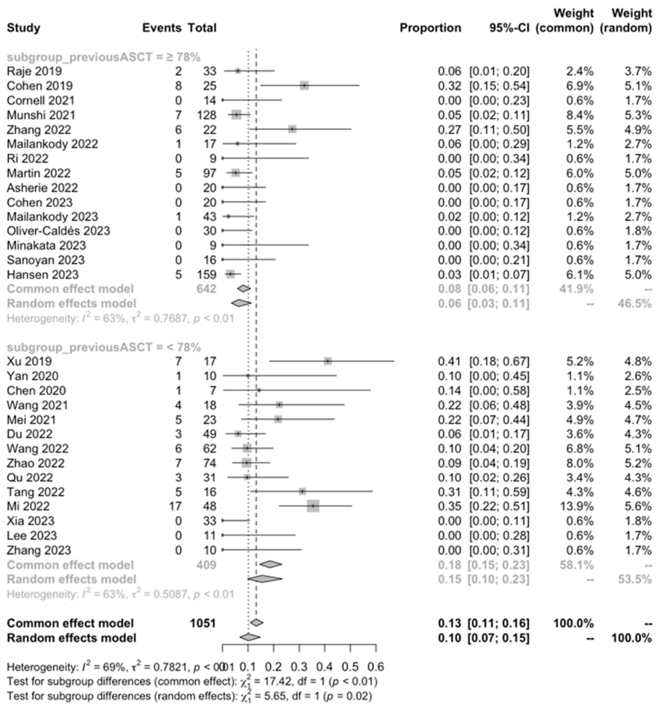
Study Events Total Proportion 95%-CI Weight (common) Weight (random)
subgroup_previousASCT = ≥ 78%
Raje 2019 2 33 0.06 [0.01; 0.20] 2.4% 3.7%
Cohen 2019 8 25 0.32 [0.15; 0.54] 6.9% 5.1%
Cornell 2021 0 14 0.00 [0.00; 0.23] 0.6% 1.7%
Munshi 2021 7 128 0.05 [0.02; 0.11] 8.4% 5.3%
Zhang 2022 6 22 0.27 [0.11; 0.50] 5.5% 4.9%
Mailankody 2022 1 17 0.06 [0.00; 0.29] 1.2% 2.7%
Ri 2022 0 9 0.00 [0.00; 0.34] 0.6% 1.7%
Martin 2022 5 97 0.05 [0.02; 0.12] 6.0% 5.0%
Asherie 2022 0 20 0.00 [0.00; 0.17] 0.6% 1.7%
Cohen 2023 0 20 0.00 [0.00; 0.17] 0.6% 1.7%
Mailankody 2023 1 43 0.02 [0.00; 0.12] 1.2% 2.7%
Oliver-Caldés 2023 0 30 0.00 [0.00; 0.12] 0.6% 1.8%
Minakata 2023 0 9 0.00 [0.00; 0.34] 0.6% 1.7%
Sanoyan 2023 0 16 0.00 [0.00; 0.21] 0.6% 1.7%
Hansen 2023 5 159 0.03 [0.01; 0.07] 6.1% 5.0%
Common effect model 642 0.08 [0.06; 0.11] 41.9% --
Random effects model 0.06 [0.03; 0.11] -- 46.5%
Heterogeneity: I^2 = 63%, τ^2 = 0.7687, p < 0.01
subgroup_previousASCT = < 78%
Xu 2019 7 17 0.41 [0.18; 0.67] 5.2% 4.8%
Yan 2020 1 10 0.10 [0.00; 0.45] 1.1% 2.6%
Chen 2020 1 7 0.14 [0.00; 0.58] 1.1% 2.5%
Wang 2021 4 18 0.22 [0.06; 0.48] 3.9% 4.5%
Mei 2021 5 23 0.22 [0.07; 0.44] 4.9% 4.7%
Du 2022 3 49 0.06 [0.01; 0.17] 3.6% 4.3%
Wang 2022 6 62 0.10 [0.04; 0.20] 6.8% 5.1%
Zhao 2022 7 74 0.09 [0.04; 0.19] 8.0% 5.2%
Qu 2022 3 31 0.10 [0.02; 0.26] 3.4% 4.3%
Tang 2022 5 16 0.31 [0.11; 0.59] 4.3% 4.6%
Mi 2022 17 48 0.35 [0.22; 0.51] 13.9% 5.6%
Xia 2023 0 33 0.00 [0.00; 0.11] 0.6% 1.8%
Lee 2023 0 11 0.00 [0.00; 0.28] 0.6% 1.7%
Zhang 2023 0 10 0.00 [0.00; 0.31] 0.6% 1.7%
Common effect model 409 0.18 [0.15; 0.23] 58.1% --
Random effects model 0.15 [0.10; 0.23] -- 53.5%
Heterogeneity: I^2 = 63%, τ^2 = 0.5087, p < 0.01
Common effect model 1051 0.13 [0.11; 0.16] 100.0% --
Random effects model 0.10 [0.07; 0.15] -- 100.0%
Heterogeneity: I^2 = 69%, τ^2 = 0.7821, p < 0 0.1 0.1 0.2 0.3 0.4 0.5 0.6
Test for subgroup differences (common effect): χ^2_1 = 17.42, df = 1 (p < 0.01)
Test for subgroup differences (random effects): χ^2_1 = 5.65, df = 1 (p = 0.02)

(D)

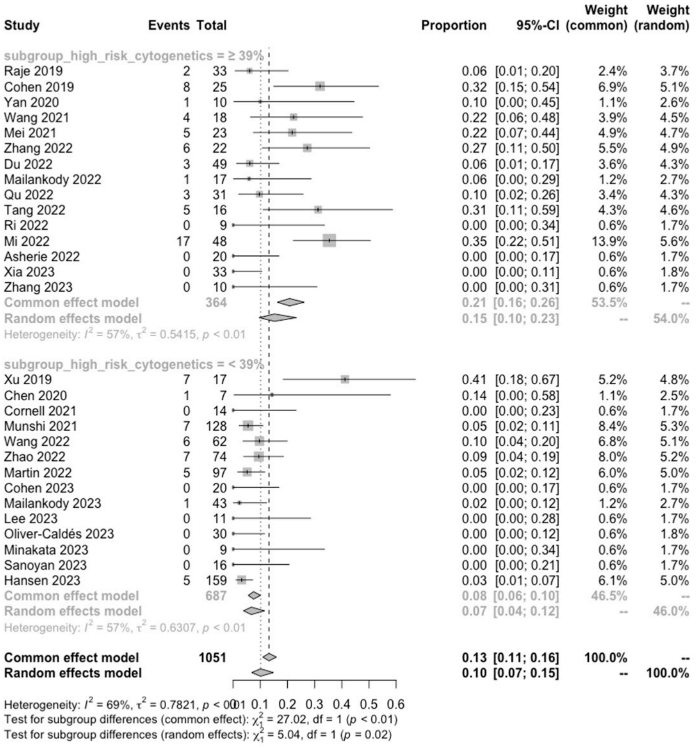
Study Events Total Proportion 95%-CI Weight (common) Weight (random)
subgroup_high_risk_cytogenetics = ≥ 39%
Raje 2019 2 33 0.06 [0.01; 0.20] 2.4% 3.7%
Cohen 2019 8 25 0.32 [0.15; 0.54] 6.9% 5.1%
Yan 2020 1 10 0.10 [0.00; 0.45] 1.1% 2.6%
Wang 2021 4 18 0.22 [0.06; 0.48] 3.9% 4.5%
Mei 2021 5 23 0.22 [0.07; 0.44] 4.9% 4.7%
Zhang 2022 6 22 0.27 [0.11; 0.50] 5.5% 4.9%
Du 2022 3 49 0.06 [0.01; 0.17] 3.6% 4.3%
Mailankody 2022 1 17 0.06 [0.00; 0.29] 1.2% 2.7%
Qu 2022 3 31 0.10 [0.02; 0.26] 3.4% 4.3%
Tang 2022 5 16 0.31 [0.11; 0.59] 4.3% 4.6%
Ri 2022 0 9 0.00 [0.00; 0.34] 0.6% 1.7%
Mi 2022 17 48 0.35 [0.22; 0.51] 13.9% 5.6%
Asherie 2022 0 20 0.00 [0.00; 0.17] 0.6% 1.7%
Xia 2023 0 33 0.00 [0.00; 0.11] 0.6% 1.8%
Zhang 2023 0 10 0.00 [0.00; 0.31] 0.6% 1.7%
Common effect model 364 0.21 [0.16; 0.26] 53.5% --
Random effects model 0.15 [0.10; 0.23] -- 54.0%
Heterogeneity: I² = 57%, τ² = 0.5415, p < 0.01
subgroup_high_risk_cytogenetics = < 39%
Xu 2019 7 17 0.41 [0.18; 0.67] 5.2% 4.8%
Chen 2020 1 7 0.14 [0.00; 0.58] 1.1% 2.5%
Cornell 2021 0 14 0.00 [0.00; 0.23] 0.6% 1.7%
Munshi 2021 7 128 0.05 [0.02; 0.11] 8.4% 5.3%
Wang 2022 6 62 0.10 [0.04; 0.20] 6.8% 5.1%
Zhao 2022 7 74 0.09 [0.04; 0.19] 8.0% 5.2%
Martin 2022 5 97 0.05 [0.02; 0.12] 6.0% 5.0%
Cohen 2023 0 20 0.00 [0.00; 0.17] 0.6% 1.7%
Mailankody 2023 1 43 0.02 [0.00; 0.12] 1.2% 2.7%
Lee 2023 0 11 0.00 [0.00; 0.28] 0.6% 1.7%
Oliver-Caldés 2023 0 30 0.00 [0.00; 0.12] 0.6% 1.8%
Minakata 2023 0 9 0.00 [0.00; 0.34] 0.6% 1.7%
Sanoyan 2023 0 16 0.00 [0.00; 0.21] 0.6% 1.7%
Hansen 2023 5 159 0.03 [0.01; 0.07] 6.1% 5.0%
Common effect model 687 0.08 [0.06; 0.10] 46.5% --
Random effects model 0.07 [0.04; 0.12] -- 46.0%
Heterogeneity: I² = 57%, τ² = 0.6307, p < 0.01
Common effect model 1051 0.13 [0.11; 0.16] 100.0% --
Random effects model 0.10 [0.07; 0.15] -- 100.0%
Heterogeneity: I² = 69%, τ² = 0.7821, p < 0.01
0 0.1 0.2 0.3 0.4 0.5 0.6
Test for subgroup differences (common effect): χ²₁ = 27.02, df = 1 (p < 0.01)
Test for subgroup differences (random effects): χ²₁ = 5.04, df = 1 (p = 0.02)

(E)

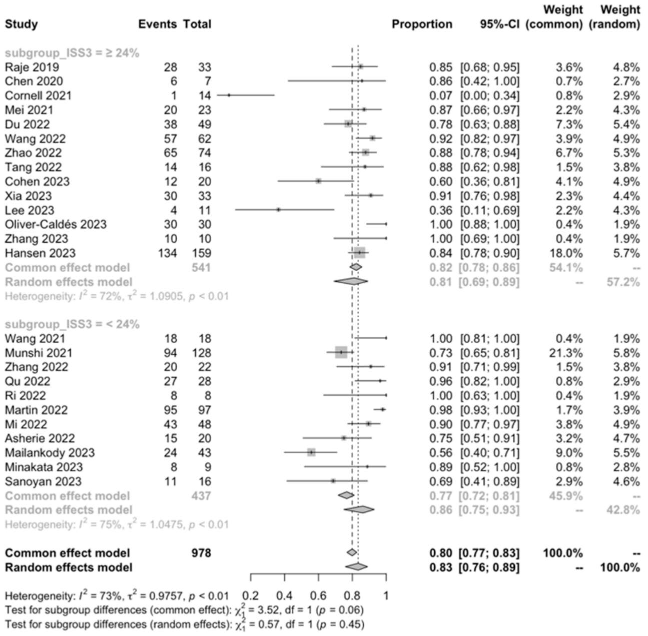
Study Events Total Proportion 95%-CI Weight (common) Weight (random)
subgroup_ISS3 = ≥ 24%
Raje 2019 28 33 0.85 [0.68; 0.95] 3.6% 4.8%
Chen 2020 6 7 0.86 [0.42; 1.00] 0.7% 2.7%
Cornell 2021 1 14 0.07 [0.00; 0.34] 0.8% 2.9%
Mei 2021 20 23 0.87 [0.66; 0.97] 2.2% 4.3%
Du 2022 38 49 0.78 [0.63; 0.88] 7.3% 5.4%
Wang 2022 57 62 0.92 [0.82; 0.97] 3.9% 4.9%
Zhao 2022 65 74 0.88 [0.78; 0.94] 6.7% 5.3%
Tang 2022 14 16 0.88 [0.62; 0.98] 1.5% 3.8%
Cohen 2023 12 20 0.60 [0.36; 0.81] 4.1% 4.9%
Xia 2023 30 33 0.91 [0.76; 0.98] 2.3% 4.4%
Lee 2023 4 11 0.36 [0.11; 0.69] 2.2% 4.3%
Oliver-Caldés 2023 30 30 1.00 [0.88; 1.00] 0.4% 1.9%
Zhang 2023 10 10 1.00 [0.69; 1.00] 0.4% 1.9%
Hansen 2023 134 159 0.84 [0.78; 0.90] 18.0% 5.7%
Common effect model 541 0.82 [0.78; 0.86] 54.1% --
Random effects model 0.81 [0.69; 0.89] -- 57.2%
Heterogeneity: I² = 72%, τ² = 1.0905, p < 0.01
subgroup_ISS3 = < 24%
Wang 2021 18 18 1.00 [0.81; 1.00] 0.4% 1.9%
Munshi 2021 94 128 0.73 [0.65; 0.81] 21.3% 5.8%
Zhang 2022 20 22 0.91 [0.71; 0.99] 1.5% 3.8%
Qu 2022 27 28 0.96 [0.82; 1.00] 0.8% 2.9%
Ri 2022 8 8 1.00 [0.63; 1.00] 0.4% 1.9%
Martin 2022 95 97 0.98 [0.93; 1.00] 1.7% 3.9%
Mi 2022 43 48 0.90 [0.77; 0.97] 3.8% 4.9%
Asherie 2022 15 20 0.75 [0.51; 0.91] 3.2% 4.7%
Mailankody 2023 24 43 0.56 [0.40; 0.71] 9.0% 5.5%
Minakata 2023 8 9 0.89 [0.52; 1.00] 0.8% 2.8%
Sanoyan 2023 11 16 0.69 [0.41; 0.89] 2.9% 4.6%
Common effect model 437 0.77 [0.72; 0.81] 45.9% --
Random effects model 0.86 [0.75; 0.93] -- 42.8%
Heterogeneity: I² = 75%, τ² = 1.0475, p < 0.01
Common effect model 978 0.80 [0.77; 0.83] 100.0% --
Random effects model 0.83 [0.76; 0.89] -- 100.0%
Heterogeneity: I² = 73%, τ² = 0.9757, p < 0.01
0.2 0.4 0.6 0.8 1
Test for subgroup differences (common effect): χ²₁ = 3.52, df = 1 (p = 0.06)
Test for subgroup differences (random effects): χ²₁ = 0.57, df = 1 (p = 0.45)

(F)

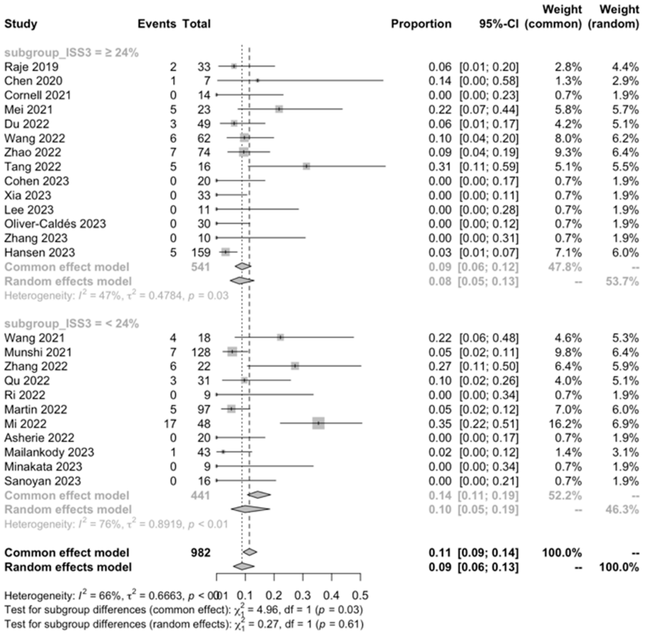
Study Events Total Proportion 95%-CI Weight (common) Weight (random)
subgroup_ISS3 = ≥ 24%
Raje 2019 2 33 0.06 [0.01; 0.20] 2.8% 4.4%
Chen 2020 1 7 0.14 [0.00; 0.58] 1.3% 2.9%
Cornell 2021 0 14 0.00 [0.00; 0.23] 0.7% 1.9%
Mei 2021 5 23 0.22 [0.07; 0.44] 5.8% 5.7%
Du 2022 3 49 0.06 [0.01; 0.17] 4.2% 5.1%
Wang 2022 6 62 0.10 [0.04; 0.20] 8.0% 6.2%
Zhao 2022 7 74 0.09 [0.04; 0.19] 9.3% 6.4%
Tang 2022 5 16 0.31 [0.11; 0.59] 5.1% 5.5%
Cohen 2023 0 20 0.00 [0.00; 0.17] 0.7% 1.9%
Xia 2023 0 33 0.00 [0.00; 0.11] 0.7% 1.9%
Lee 2023 0 11 0.00 [0.00; 0.28] 0.7% 1.9%
Oliver-Caldés 2023 0 30 0.00 [0.00; 0.12] 0.7% 1.9%
Zhang 2023 0 10 0.00 [0.00; 0.31] 0.7% 1.9%
Hansen 2023 5 159 0.03 [0.01; 0.07] 7.1% 6.0%
Common effect model 541 0.09 [0.06; 0.12] 47.8% --
Random effects model 0.08 [0.05; 0.13] -- 53.7%
Heterogeneity: I² = 47%, τ² = 0.4784, p = 0.03
subgroup_ISS3 = < 24%
Wang 2021 4 18 0.22 [0.06; 0.48] 4.6% 5.3%
Munshi 2021 7 128 0.05 [0.02; 0.11] 9.8% 6.4%
Zhang 2022 6 22 0.27 [0.11; 0.50] 6.4% 5.9%
Qu 2022 3 31 0.10 [0.02; 0.26] 4.0% 5.1%
Ri 2022 0 9 0.00 [0.00; 0.34] 0.7% 1.9%
Martin 2022 5 97 0.05 [0.02; 0.12] 7.0% 6.0%
Mi 2022 17 48 0.35 [0.22; 0.51] 16.2% 6.9%
Asherie 2022 0 20 0.00 [0.00; 0.17] 0.7% 1.9%
Mailankody 2023 1 43 0.02 [0.00; 0.12] 1.4% 3.1%
Minakata 2023 0 9 0.00 [0.00; 0.34] 0.7% 1.9%
Sanoyan 2023 0 16 0.00 [0.00; 0.21] 0.7% 1.9%
Common effect model 441 0.14 [0.11; 0.19] 52.2% --
Random effects model 0.10 [0.05; 0.19] -- 46.3%
Heterogeneity: I² = 76%, τ² = 0.8919, p < 0.01
Common effect model 982 0.11 [0.09; 0.14] 100.0% --
Random effects model 0.09 [0.06; 0.13] -- 100.0%
Heterogeneity: I² = 66%, τ² = 0.6663, p < 0.01
0 0.1 0.2 0.3 0.4 0.5
Test for subgroup differences (common effect): χ²₁ = 4.96, df = 1 (p = 0.03)
Test for subgroup differences (random effects): χ²₁ = 0.27, df = 1 (p = 0.61)

(G)

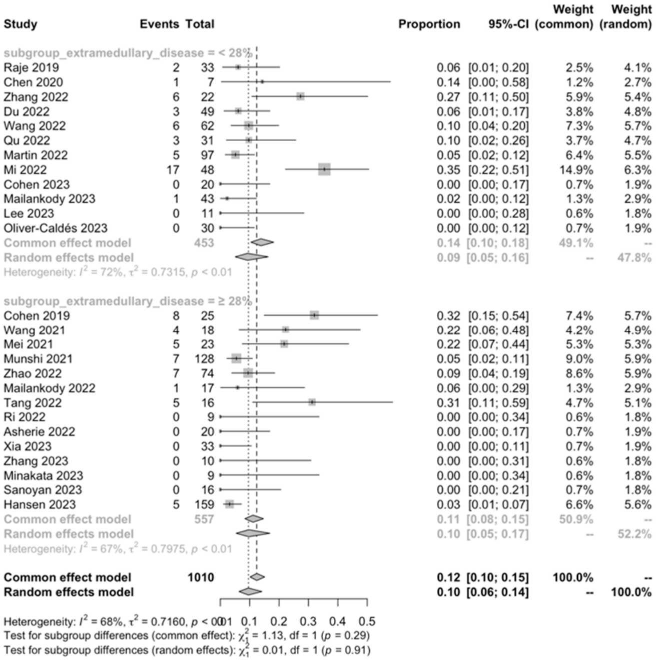
Study Events Total Proportion 95%-CI Weight (common) Weight (random)
subgroup_extramedullary_disease = < 28%
Raje 2019 2 33 0.06 [0.01; 0.20] 2.5% 4.1%
Chen 2020 1 7 0.14 [0.00; 0.58] 1.2% 2.7%
Zhang 2022 6 22 0.27 [0.11; 0.50] 5.9% 5.4%
Du 2022 3 49 0.06 [0.01; 0.17] 3.8% 4.8%
Wang 2022 6 62 0.10 [0.04; 0.20] 7.3% 5.7%
Qu 2022 3 31 0.10 [0.02; 0.26] 3.7% 4.7%
Martin 2022 5 97 0.05 [0.02; 0.12] 6.4% 5.5%
Mi 2022 17 48 0.35 [0.22; 0.51] 14.9% 6.3%
Cohen 2023 0 20 0.00 [0.00; 0.17] 0.7% 1.9%
Mailankody 2023 1 43 0.02 [0.00; 0.12] 1.3% 2.9%
Lee 2023 0 11 0.00 [0.00; 0.28] 0.6% 1.8%
Oliver-Caldés 2023 0 30 0.00 [0.00; 0.12] 0.7% 1.9%
Common effect model 453 0.14 [0.10; 0.18] 49.1% --
Random effects model 0.09 [0.05; 0.16] -- 47.8%
Heterogeneity: I² = 72%, τ² = 0.7315, p < 0.01
subgroup_extramedullary_disease = ≥ 28%
Cohen 2019 8 25 0.32 [0.15; 0.54] 7.4% 5.7%
Wang 2021 4 18 0.22 [0.06; 0.48] 4.2% 4.9%
Mei 2021 5 23 0.22 [0.07; 0.44] 5.3% 5.3%
Munshi 2021 7 128 0.05 [0.02; 0.11] 9.0% 5.9%
Zhao 2022 7 74 0.09 [0.04; 0.19] 8.6% 5.9%
Mailankody 2022 1 17 0.06 [0.00; 0.29] 1.3% 2.9%
Tang 2022 5 16 0.31 [0.11; 0.59] 4.7% 5.1%
Ri 2022 0 9 0.00 [0.00; 0.34] 0.6% 1.8%
Asherie 2022 0 20 0.00 [0.00; 0.17] 0.7% 1.9%
Xia 2023 0 33 0.00 [0.00; 0.11] 0.7% 1.9%
Zhang 2023 0 10 0.00 [0.00; 0.31] 0.6% 1.8%
Minakata 2023 0 9 0.00 [0.00; 0.34] 0.6% 1.8%
Sanoyan 2023 0 16 0.00 [0.00; 0.21] 0.7% 1.8%
Hansen 2023 5 159 0.03 [0.01; 0.07] 6.6% 5.6%
Common effect model 557 0.11 [0.08; 0.15] 50.9% --
Random effects model 0.10 [0.05; 0.17] -- 52.2%
Heterogeneity: I² = 67%, τ² = 0.7975, p < 0.01
Common effect model 1010 0.12 [0.10; 0.15] 100.0% --
Random effects model 0.10 [0.06; 0.14] -- 100.0%
Heterogeneity: I² = 68%, τ² = 0.7160, p < 0.01
0 0.1 0.2 0.3 0.4 0.5
Test for subgroup differences (common effect): χ²₁ = 1.13, df = 1 (p = 0.29)
Test for subgroup differences (random effects): χ²₁ = 0.01, df = 1 (p = 0.91)

(H)

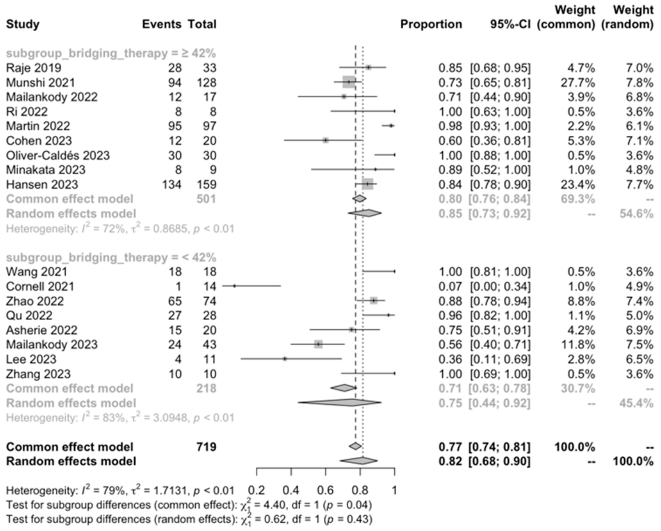
Study Events Total Proportion 95%-CI Weight (common) Weight (random)
subgroup_bridging_therapy = ≥ 42%
Raje 2019 28 33 0.85 [0.68; 0.95] 4.7% 7.0%
Munshi 2021 94 128 0.73 [0.65; 0.81] 27.7% 7.8%
Mailankody 2022 12 17 0.71 [0.44; 0.90] 3.9% 6.8%
Ri 2022 8 8 1.00 [0.63; 1.00] 0.5% 3.6%
Martin 2022 95 97 0.98 [0.93; 1.00] 2.2% 6.1%
Cohen 2023 12 20 0.60 [0.36; 0.81] 5.3% 7.1%
Oliver-Caldés 2023 30 30 1.00 [0.88; 1.00] 0.5% 3.6%
Minakata 2023 8 9 0.89 [0.52; 1.00] 1.0% 4.8%
Hansen 2023 134 159 0.84 [0.78; 0.90] 23.4% 7.7%
Common effect model 501 0.80 [0.76; 0.84] 69.3% --
Random effects model 0.85 [0.73; 0.92] -- 54.6%
Heterogeneity: I² = 72%, τ² = 0.8685, p < 0.01
subgroup_bridging_therapy = < 42%
Wang 2021 18 18 1.00 [0.81; 1.00] 0.5% 3.6%
Cornell 2021 1 14 0.07 [0.00; 0.34] 1.0% 4.9%
Zhao 2022 65 74 0.88 [0.78; 0.94] 8.8% 7.4%
Qu 2022 27 28 0.96 [0.82; 1.00] 1.1% 5.0%
Asherie 2022 15 20 0.75 [0.51; 0.91] 4.2% 6.9%
Mailankody 2023 24 43 0.56 [0.40; 0.71] 11.8% 7.5%
Lee 2023 4 11 0.36 [0.11; 0.69] 2.8% 6.5%
Zhang 2023 10 10 1.00 [0.69; 1.00] 0.5% 3.6%
Common effect model 218 0.71 [0.63; 0.78] 30.7% --
Random effects model 0.75 [0.44; 0.92] -- 45.4%
Heterogeneity: I² = 83%, τ² = 3.0948, p < 0.01
Common effect model 719 0.77 [0.74; 0.81] 100.0% --
Random effects model 0.82 [0.68; 0.90] -- 100.0%
Heterogeneity: I² = 79%, τ² = 1.7131, p < 0.01
0.2 0.4 0.6 0.8 1
Test for subgroup differences (common effect): χ²₁ = 4.40, df = 1 (p = 0.04)
Test for subgroup differences (random effects): χ²₁ = 0.62, df = 1 (p = 0.43)

(I)

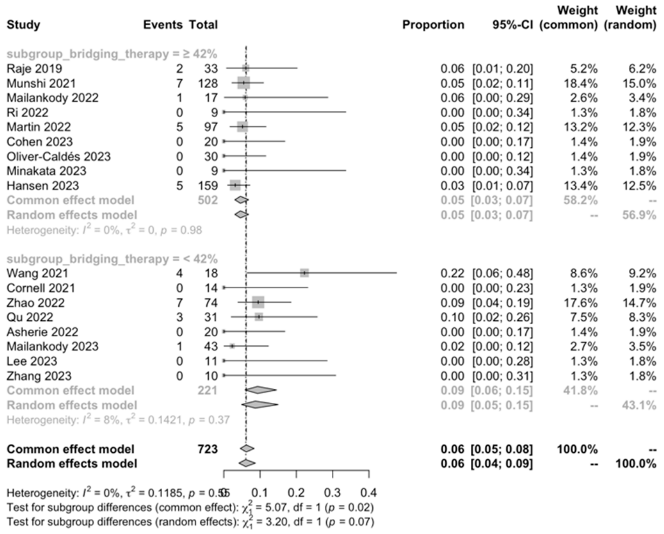
Study Events Total Proportion 95%-CI Weight (common) Weight (random)
subgroup_bridging_therapy = ≥ 42%
Raje 2019 2 33 0.06 [0.01; 0.20] 5.2% 6.2%
Munshi 2021 7 128 0.05 [0.02; 0.11] 18.4% 15.0%
Mailankody 2022 1 17 0.06 [0.00; 0.29] 2.6% 3.4%
Ri 2022 0 9 0.00 [0.00; 0.34] 1.3% 1.8%
Martin 2022 5 97 0.05 [0.02; 0.12] 13.2% 12.3%
Cohen 2023 0 20 0.00 [0.00; 0.17] 1.4% 1.9%
Oliver-Caldés 2023 0 30 0.00 [0.00; 0.12] 1.4% 1.9%
Minakata 2023 0 9 0.00 [0.00; 0.34] 1.3% 1.8%
Hansen 2023 5 159 0.03 [0.01; 0.07] 13.4% 12.5%
Common effect model 502 0.05 [0.03; 0.07] 58.2% --
Random effects model 0.05 [0.03; 0.07] -- 56.9%
Heterogeneity: I² = 0%, τ² = 0, p = 0.98
subgroup_bridging_therapy = < 42%
Wang 2021 4 18 0.22 [0.06; 0.48] 8.6% 9.2%
Cornell 2021 0 14 0.00 [0.00; 0.23] 1.3% 1.9%
Zhao 2022 7 74 0.09 [0.04; 0.19] 17.6% 14.7%
Qu 2022 3 31 0.10 [0.02; 0.26] 7.5% 8.3%
Asherie 2022 0 20 0.00 [0.00; 0.17] 1.4% 1.9%
Mailankody 2023 1 43 0.02 [0.00; 0.12] 2.7% 3.5%
Lee 2023 0 11 0.00 [0.00; 0.28] 1.3% 1.8%
Zhang 2023 0 10 0.00 [0.00; 0.31] 1.3% 1.8%
Common effect model 221 0.09 [0.06; 0.15] 41.8% --
Random effects model 0.09 [0.05; 0.15] -- 43.1%
Heterogeneity: I² = 8%, τ² = 0.1421, p = 0.37
Common effect model 723 0.06 [0.05; 0.08] 100.0% --
Random effects model 0.06 [0.04; 0.09] -- 100.0%
0.1 0.2 0.3 0.4
Heterogeneity: I² = 0%, τ² = 0.1185, p = 0.56
Test for subgroup differences (common effect): χ²₁ = 5.07, df = 1 (p = 0.02)
Test for subgroup differences (random effects): χ²₁ = 3.20, df = 1 (p = 0.07)

(J)

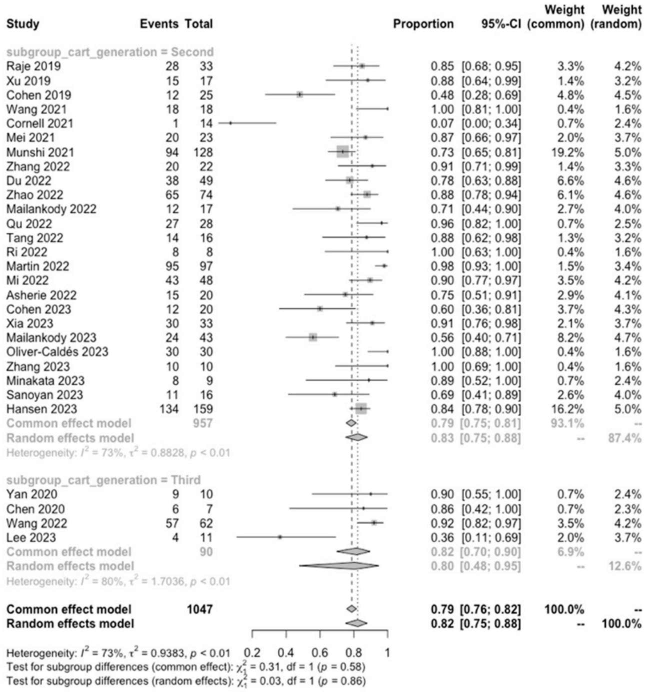
Study Events Total Proportion 95%-CI Weight (common) Weight (random)
subgroup_cart_generation = Second
Raje 2019 28 33 0.85 [0.68; 0.95] 3.3% 4.2%
Xu 2019 15 17 0.88 [0.64; 0.99] 1.4% 3.2%
Cohen 2019 12 25 0.48 [0.28; 0.69] 4.8% 4.5%
Wang 2021 18 18 1.00 [0.81; 1.00] 0.4% 1.6%
Cornell 2021 1 14 0.07 [0.00; 0.34] 0.7% 2.4%
Mei 2021 20 23 0.87 [0.66; 0.97] 2.0% 3.7%
Munshi 2021 94 128 0.73 [0.65; 0.81] 19.2% 5.0%
Zhang 2022 20 22 0.91 [0.71; 0.99] 1.4% 3.3%
Du 2022 38 49 0.78 [0.63; 0.88] 6.6% 4.6%
Zhao 2022 65 74 0.88 [0.78; 0.94] 6.1% 4.6%
Mailankody 2022 12 17 0.71 [0.44; 0.90] 2.7% 4.0%
Qu 2022 27 28 0.96 [0.82; 1.00] 0.7% 2.5%
Tang 2022 14 16 0.88 [0.62; 0.98] 1.3% 3.2%
Ri 2022 8 8 1.00 [0.63; 1.00] 0.4% 1.6%
Martin 2022 95 97 0.98 [0.93; 1.00] 1.5% 3.4%
Mi 2022 43 48 0.90 [0.77; 0.97] 3.5% 4.2%
Asherie 2022 15 20 0.75 [0.51; 0.91] 2.9% 4.1%
Cohen 2023 12 20 0.60 [0.36; 0.81] 3.7% 4.3%
Xia 2023 30 33 0.91 [0.76; 0.98] 2.1% 3.7%
Mailankody 2023 24 43 0.56 [0.40; 0.71] 8.2% 4.7%
Oliver-Caldés 2023 30 30 1.00 [0.88; 1.00] 0.4% 1.6%
Zhang 2023 10 10 1.00 [0.69; 1.00] 0.4% 1.6%
Minakata 2023 8 9 0.89 [0.52; 1.00] 0.7% 2.4%
Sanoyan 2023 11 16 0.69 [0.41; 0.89] 2.6% 4.0%
Hansen 2023 134 159 0.84 [0.78; 0.90] 16.2% 5.0%
Common effect model 957 0.79 [0.75; 0.81] 93.1% --
Random effects model 0.83 [0.75; 0.88] -- 87.4%
Heterogeneity: I² = 73%, τ² = 0.8828, p < 0.01
subgroup_cart_generation = Third
Yan 2020 9 10 0.90 [0.55; 1.00] 0.7% 2.4%
Chen 2020 6 7 0.86 [0.42; 1.00] 0.7% 2.3%
Wang 2022 57 62 0.92 [0.82; 0.97] 3.5% 4.2%
Lee 2023 4 11 0.36 [0.11; 0.69] 2.0% 3.7%
Common effect model 90 0.82 [0.70; 0.90] 6.9% --
Random effects model 0.80 [0.48; 0.95] -- 12.6%
Heterogeneity: I² = 80%, τ² = 1.7036, p < 0.01
Common effect model 1047 0.79 [0.76; 0.82] 100.0% --
Random effects model 0.82 [0.75; 0.88] -- 100.0%
Heterogeneity: I² = 73%, τ² = 0.9383, p < 0.01
0.2 0.4 0.6 0.8 1
Test for subgroup differences (common effect): χ²₁ = 0.31, df = 1 (p = 0.58)
Test for subgroup differences (random effects): χ²₁ = 0.03, df = 1 (p = 0.86)

(K)

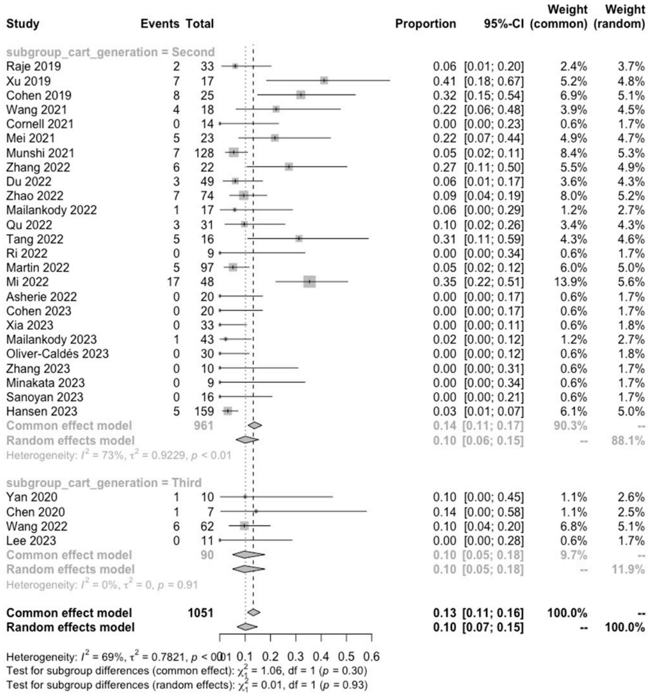
Study Events Total Proportion 95%-CI Weight (common) Weight (random)
subgroup_cart_generation = Second
Raje 2019 2 33 0.06 [0.01; 0.20] 2.4% 3.7%
Xu 2019 7 17 0.41 [0.18; 0.67] 5.2% 4.8%
Cohen 2019 8 25 0.32 [0.15; 0.54] 6.9% 5.1%
Wang 2021 4 18 0.22 [0.06; 0.48] 3.9% 4.5%
Cornell 2021 0 14 0.00 [0.00; 0.23] 0.6% 1.7%
Mei 2021 5 23 0.22 [0.07; 0.44] 4.9% 4.7%
Munshi 2021 7 128 0.05 [0.02; 0.11] 8.4% 5.3%
Zhang 2022 6 22 0.27 [0.11; 0.50] 5.5% 4.9%
Du 2022 3 49 0.06 [0.01; 0.17] 3.6% 4.3%
Zhao 2022 7 74 0.09 [0.04; 0.19] 8.0% 5.2%
Mailankody 2022 1 17 0.06 [0.00; 0.29] 1.2% 2.7%
Qu 2022 3 31 0.10 [0.02; 0.26] 3.4% 4.3%
Tang 2022 5 16 0.31 [0.11; 0.59] 4.3% 4.6%
Ri 2022 0 9 0.00 [0.00; 0.34] 0.6% 1.7%
Martin 2022 5 97 0.05 [0.02; 0.12] 6.0% 5.0%
Mi 2022 17 48 0.35 [0.22; 0.51] 13.9% 5.6%
Asherie 2022 0 20 0.00 [0.00; 0.17] 0.6% 1.7%
Cohen 2023 0 20 0.00 [0.00; 0.17] 0.6% 1.7%
Xia 2023 0 33 0.00 [0.00; 0.11] 0.6% 1.8%
Mailankody 2023 1 43 0.02 [0.00; 0.12] 1.2% 2.7%
Oliver-Caldés 2023 0 30 0.00 [0.00; 0.12] 0.6% 1.8%
Zhang 2023 0 10 0.00 [0.00; 0.31] 0.6% 1.7%
Minakata 2023 0 9 0.00 [0.00; 0.34] 0.6% 1.7%
Sanoyan 2023 0 16 0.00 [0.00; 0.21] 0.6% 1.7%
Hansen 2023 5 159 0.03 [0.01; 0.07] 6.1% 5.0%
Common effect model 961 0.14 [0.11; 0.17] 90.3% --
Random effects model 0.10 [0.06; 0.15] -- 88.1%
Heterogeneity: I² = 73%, τ² = 0.9229, p < 0.01
subgroup_cart_generation = Third
Yan 2020 1 10 0.10 [0.00; 0.45] 1.1% 2.6%
Chen 2020 1 7 0.14 [0.00; 0.58] 1.1% 2.5%
Wang 2022 6 62 0.10 [0.04; 0.20] 6.8% 5.1%
Lee 2023 0 11 0.00 [0.00; 0.28] 0.6% 1.7%
Common effect model 90 0.10 [0.05; 0.18] 9.7% --
Random effects model 0.10 [0.05; 0.18] -- 11.9%
Heterogeneity: I² = 0%, τ² = 0, p = 0.91
Common effect model 1051 0.13 [0.11; 0.16] 100.0% --
Random effects model 0.10 [0.07; 0.15] -- 100.0%
Heterogeneity: I² = 69%, τ² = 0.7821, p < 0.01
0.1 0.2 0.3 0.4 0.5 0.6
Test for subgroup differences (common effect): χ²₁ = 1.06, df = 1 (p = 0.30)
Test for subgroup differences (random effects): χ²₁ = 0.01, df = 1 (p = 0.93)

(L)

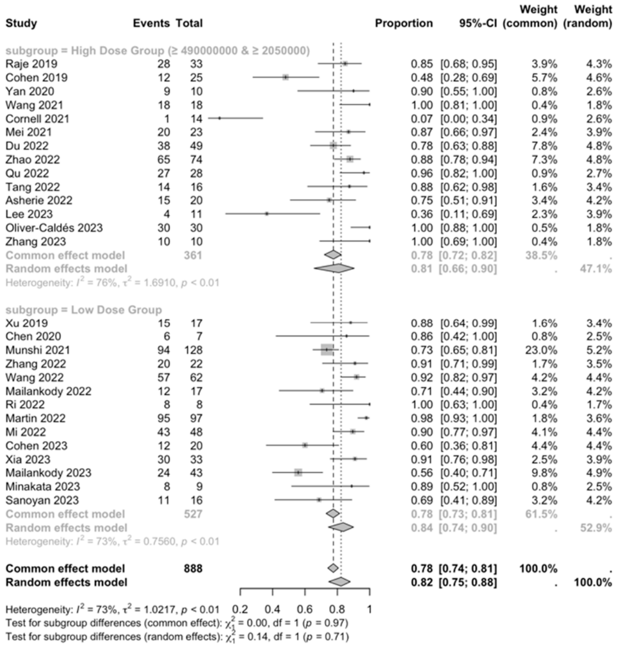

(M)

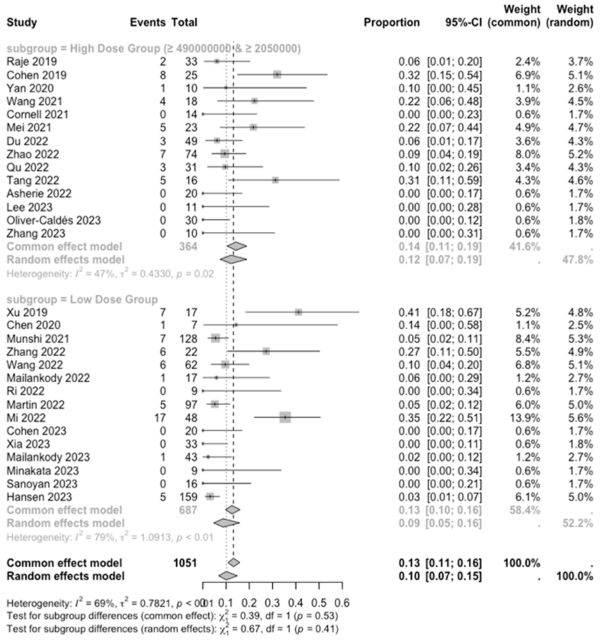

(N)

Figure S3. Subgroup analysis : **(A)** grade ≥ 3 CRS, prior antimyeloma regimens; **(B)** grade ≥ 3 CRS, prior exposure to BCMA therapy; **(C)** ORR, prior ASCT; **(D)** grade ≥ 3 CRS, prior ASCT; **(E)** grade ≥ 3 CRS, high-risk cytogenetics; **(F)** ORR, ISS stage 3; **(G)** grade ≥ 3 CRS, ISS stage 3; **(H)** grade ≥ 3 CRS, extramedullary disease; **(I)** ORR, bridging therapy; **(J)** grade ≥ 3 CRS, bridging therapy; **(K)** ORR, CAR-T generation; **(L)** grade ≥ 3 CRS, CAR-T generation; **(M)** ORR, upper infusion threshold; **(N)** grade ≥ 3 CRS, upper infusion threshold.
